# Novel Drug Targets and Emerging Pharmacotherapies in Neuropathic Pain

**DOI:** 10.3390/pharmaceutics15071799

**Published:** 2023-06-23

**Authors:** Jurga Bernatoniene, Arunas Sciupokas, Dalia Marija Kopustinskiene, Kestutis Petrikonis

**Affiliations:** 1 Department of Drug Technology and Social Pharmacy, Faculty of Pharmacy, Medical Academy, Lithuanian University of Health Sciences, Sukileliu pr. 13, LT-50161 Kaunas, Lithuania; 2 Institute of Pharmaceutical Technologies, Faculty of Pharmacy, Medical Academy, Lithuanian University of Health Sciences, Sukileliu pr. 13, LT-50161 Kaunas, Lithuania; daliamarija.kopustinskiene@lsmuni.lt; 3 Pain Clinic, Lithuanian University of Health Sciences Hospital Kauno Klinikos, Eivenių Str. 2, LT-50009 Kaunas, Lithuania; arunas.sciupokas@lsmu.lt; 4 Department of Neurology, Lithuanian University of Health Sciences, Eivenių Str. 2, LT-50009 Kaunas, Lithuania; kestutis.petrikonis@lsmu.lt

**Keywords:** neuropathic pain, therapy, tricyclic antidepressants, gabapentinoids, ambroxol, cannabidiol, N-acetyl-L-cysteine

## Abstract

Neuropathic pain is a debilitating condition characterized by abnormal signaling within the nervous system, resulting in persistent and often intense sensations of pain. It can arise from various causes, including traumatic nerve injury, neuropathy, and certain diseases. We present an overview of current and emerging pharmacotherapies for neuropathic pain, focusing on novel drug targets and potential therapeutic agents. Current pharmacotherapies, including tricyclic antidepressants, gabapentinoids, and serotonin norepinephrine re-uptake inhibitors, are discussed, as are emerging treatments, such as ambroxol, cannabidiol, and N-acetyl-L-cysteine. Additionally, the article highlights the need for further research in this field to identify new targets and develop more effective and targeted therapies for neuropathic pain management.

## 1. Introduction

When sensory division of the nervous system is damaged or malfunctioning, it can cause a painful condition known as neuropathic pain [1]. Generally, according to these mechanisms [2,3], pain is classified into three categories: nociceptive pain, which results from an acute injury and subsides as the injury heals; neuropathic pain, which is caused by disease or damage to the sensory structures of the peripheral and/or central nervous system [4]; and nociplastic pain, which arises from altered nociception, despite there being no clear evidence of actual or threatened tissue damage causing the activation of peripheral nociceptors, or of disease or lesion of the somatosensory system causing the pain [3,5]. In some classifications, there is a fourth pain category: inflammatory pain [3]. Thus, neuropathic pain, in contrast to nociceptive pain, which originates from damaged tissues and inflammation, is typically the result of aberrant signaling inside neurons [1].

Pro-inflammatory cytokines, such as interleukin-1b (IL-1b), which are released by immune cells, microglia, and astroglia in the spinal cord, play a critical role in the etiology and mechanisms of neuropathic pain [6]. In addition, inflammation induces cyclo-oxygenase-2 (COX-2) expression, thus enhancing prostaglandin (PGE) synthesis [7]. The potential causes of neuropathic pain include nerve compression, trauma to the nervous system, diabetic neuropathy, and post-herpetic neuralgia [1,4].

Neuropathic pain progresses and typically turns into chronicity near to the very beginning of the pain process [1]. Most patients with neuropathic pain complain of ongoing or intermittent spontaneous pain. Tingling, numbness, a perception of burning, and sharp pain are most common symptoms [8,9]. Dysesthesia refers to an atypical sensation that can be severe and/or accompanied by pain. In contrast, paresthesia refers to altered sensations, such as numbness, tingling, or the sensation of “pins and needles.” Paresthesia is typically temporary and does not cause pain [8,9]. We can often find various combinations of these sensory cues. Increased sensitivity or its loss in the affected area may also occur. Sometimes, movement or touch could increase the pain, though it can also be rather persistent, even long after the initial injury heals [8,9]. The damaged nerves keep sending signals to the brain, leading to the continuous sensation of pain, due to peripheral and central sensitization, which are related to changes in ion channels, the stimulation of immune cells, substances produced by glial cells, and the modulation of gene expression patterns [1].

Neuropathic pain is clinically characterized by hyperalgesia (increased painfulness) of the affected body part’s innervated area and allodynia (painful response to non-painful stimuli). Bedside tests are helpful to identify evoked pain and sensory abnormalities. Lightly brushing the site of spontaneous pain with a piece of cotton wool may result in pain or an unpleasant sensation, demonstrating allodynia [10]. Hyperalgesia can be assessed with sufficiently sharp pin prick testing over the affected site, and if a patient reports exaggerated pain, this finding would suggest the presence of hyperalgesia. Other sensory phenomena include hyperpathia (increased reaction to a series of stimuli with subsequent prolongation of painful aftersensations when the stimulus is removed) and delocalization (a stimulus in one area produces pain in another area) [10]. Neuropathic pain questionnaires may be used as screening tools, and these tools include the neuropathic pain scale, the Leeds assessment of neuropathic symptoms and signs (LANSS), a neuropathic pain questionnaire, painDETECT, ID-pain, and the Douleur neuropathique (DN4) [10].

Neuropathic pain mechanisms can be partly effectively inhibited and modulated by substances possessing anti-inflammatory, immune response-regulating, and anti-oxidant properties. Also, it is possible to alleviate pain by modulating nerve signals with medications, such as antidepressants, anticonvulsants, and opioids [8,9]. Physical therapy with a special focus on mechanical nerve movements and conditions and vibration stimulus, as well as nerve blocks and transcutaneous electrical nerve stimulation (TENS), show promise as novel approaches in the treatment of neuropathic pain [8,9].

## 2. Drug Targets in Neuropathic Pain

**Figure 1:**
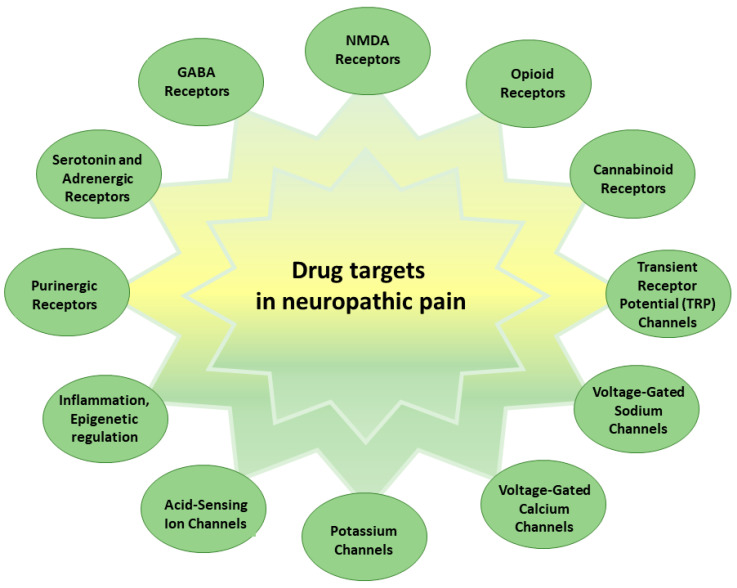
Receptors, channels, and processes that are involved in neuropathic pain.

Neuropathic pain is also characterized by the dysregulation of certain ion channels, receptors, and processes [11] (Figure 1).

NMDA receptors are a subtype of glutamate receptors that play a critical role in synaptic plasticity, learning, and memory [12,13]. In the context of neuropathic pain, NMDA receptors are known to contribute to the phenomenon of central sensitization [12,13]. Following nerve injury, the activation of NMDA receptors in the spinal cord and brain becomes enhanced, leading to an increased influx of calcium ions and the amplification of pain signals [12,13]. This heightened NMDA receptor activity promotes the establishment and maintenance of chronic pain states. Modulating NMDA receptor activity is, thus, a target for pharmacological interventions in neuropathic pain management [12,13].

Opioid receptors, including mu, delta, and kappa receptors, are distributed throughout the nervous system and are involved in pain modulation [14,15,16,17]. Endogenous opioids, such as endorphins and enkephalins, bind to opioid receptors to inhibit pain transmission. In neuropathic pain, alterations in opioid receptor expression and function can occur, resulting in reduced endogenous opioid effectiveness and diminished response to exogenous opioids, leading, in turn, to decreased pain relief [14,15,16,17]. Opioid receptor desensitization and downregulation contribute to the development of opioid tolerance and may limit the long-term efficacy of opioid-based therapies in treating neuropathic pain [14,15,16,17].

The endocannabinoid system, which consists of the cannabinoid receptors CB1 and CB2, along with endogenous ligands (endocannabinoids), plays a modulatory role in pain perception [18,19,20,21]. CB1 receptors are predominantly found in the central nervous system, while CB2 receptors are primarily located in peripheral immune cells [18,19,20,21]. Activation of cannabinoid receptors can have analgesic effects through various mechanisms, including modulation of neuronal excitability, inhibition of neurotransmitter release, and attenuation of neuroinflammation [18,19,20,21]. The endocannabinoid system has emerged as a potential target for developing cannabinoid-based medications to alleviate neuropathic pain.

Transient Receptor Potential (TRP) channels are a diverse family of ion channels involved in detecting and transmitting pain signals. Several TRP channels, such as TRPV1 (vanilloid receptor), TRPA1 (ankyrin receptor), and TRPM8 (menthol receptor), are implicated in neuropathic pain [22,23,24,25]. TRPV1 channels are activated by heat, inflammatory mediators, and capsaicin, and contribute to hypersensitivity and thermal pain in neuropathic conditions [23]. TRPA1 channels are involved in nociceptive responses to cold and chemical irritants, and their activation can enhance pain sensitivity. TRPM8 channels, which are predominantly expressed in sensory neurons, are activated by cold temperatures and play a role in cold allodynia and hyperalgesia [23]. Modulating TRP channel activity holds promise for developing novel therapeutics that target neuropathic pain [22,23,24,25].

Gamma–aminobutyric acid (GABA) receptors mediate inhibitory neurotransmission in the central nervous system [26]. GABAergic inhibition is crucial to maintaining a balance between excitation and inhibition in pain processing. Alterations in GABA receptor function, including changes in receptor subunit composition, can disrupt inhibitory signaling in neuropathic pain [26]. Decreased GABAergic inhibition and impaired GABA receptor-mediated synaptic transmission can contribute to the development and maintenance of hyperexcitability and increased pain sensitivity [26]. Enhancing GABAergic transmission or targeting specific GABA receptor subtypes may have therapeutic potential for alleviating neuropathic pain.

Serotonin (5-hydroxytryptamine, 5-HT) receptors, particularly the 5-HT3 and 5-HT1A subtypes, play a role in pain modulation [27,28]. The 5-HT3 receptor is involved in the transmission of pain signals in the spinal cord [29,30,31]. The 5HT1A heteroreceptors are found on neurons distinct from those that release serotonin (5-HT). These heteroreceptors play a crucial role in modulating pain transmission by regulating the activity of other neurotransmitter systems. When activated, the 5HT1A heteroreceptors inhibit the release of various neurotransmitters involved in pain processing, such as substance P and glutamate. By reducing the release of these pain-related neurotransmitters, the 5HT1A heteroreceptors contribute to the alleviation of pain [29,30,31]. In contrast, the 5HT1A autoreceptors are located on serotonin-releasing neurons [32,33,34]. Their primary function is to regulate the synthesis and release of serotonin. When serotonin is released, it binds to the autoreceptors, leading to a negative feedback inhibition. Activation of the 5HT1A autoreceptors results in a decrease in further serotonin release, helping to maintain optimal serotonin levels in the synaptic cleft [32,33,34]. Since serotonin is involved in inhibiting pain signals, the modulation of serotonin availability through the activation of 5HT1A autoreceptors indirectly influences pain transmission [32,33,34]. Thus, the 5HT1A heteroreceptors directly participate in pain modulation by inhibiting the release of pain-related neurotransmitters [32,33,34]. On the other hand, the 5HT1A autoreceptors regulate the synthesis and release of serotonin, indirectly affecting pain transmission by modulating serotonin availability [32,33,34]. Thus, both receptor types contribute to the overall regulation of pain perception, albeit through different mechanisms. Dysregulation of serotonin signaling was in neuropathic pain conditions, and medications targeting serotonin receptors show efficacy in managing neuropathic pain symptoms [29,35]. Selective serotonin re-uptake inhibitors (SSRIs) and serotonin–norepinephrine re-uptake inhibitors (SNRIs) are commonly used medications that modulate serotonin levels and could provide pain relief [27,28].

The noradrenergic system plays a vital role in modulating neuropathic pain, and its dysregulation can contribute to the development and persistence of this condition [36,37]. Noradrenergic neurons release noradrenaline as a neurotransmitter, which originates from the locus coeruleus in the brainstem and projects to various regions, including the spinal cord [36,38,39]. Following nerve injury, changes in noradrenergic neuron activity and function occur, leading to alterations in noradrenaline release [36,37,38,39]. In the spinal cord, noradrenaline can modulate pain signaling by acting on adrenergic receptors [37,40]. Alpha–adrenergic receptors inhibit pain neurotransmitter release and reduce the excitability of pain-sensing neurons, resulting in analgesic effects [37,40]. Beta–adrenergic receptors, with different subtypes having varied effects, can either enhance pain transmission or exert analgesic effects by modulating pain neuron activity [37,40]. Additionally, the noradrenergic system interacts with other neurotransmitter systems involved in pain processing, such as serotonin and opioids [36,39,40]. These interactions contribute to the overall modulation of pain perception and influence the development and maintenance of neuropathic pain [36,39,40]. Understanding the role of the noradrenergic system provides insights into potential therapeutic targets, such as enhancing noradrenergic signaling through medications, like norepinephrine re-uptake inhibitors or alpha-2 adrenergic receptor agonists, which show efficacy in reducing neuropathic pain symptoms [36,37,40].

Purinergic receptors, which encompass both P1 (adenosine) and P2 receptors, respond to various purine nucleotides, including ATP and adenosine [41,42]. The activation of purinergic receptors, particularly P2X receptors, can contribute to neuropathic pain by enhancing pain transmission, promoting neuroinflammation, and modulating neuronal excitability [41,42]. On the other hand, adenosine receptors specifically bind and respond to adenosine, which can have analgesic and anti-inflammatory effects [41,43,44]. Activation of adenosine receptors, particularly the A1 and A2A subtypes, was showed to inhibit pain transmission and reduce inflammation associated with neuropathic pain [41,44,45]. Therefore, while both purinergic receptors and adenosine receptors are involved in neuropathic pain, their specific mechanisms and effects may differ [42]. Purinergic receptors, including P2X receptors, are involved in the amplification of pain signals and neuroinflammation, while adenosine receptors, especially A1 and A2A receptors, have analgesic and anti-inflammatory properties [41,42,43]. Activation of adenosine receptors inhibits the release of excitatory neurotransmitters, such as glutamate, and dampens neuronal excitability [41,43]. Adenosine receptor agonists or enhancing adenosine levels in the nervous system demonstrated analgesic effects in various neuropathic pain models [41,43]. Targeting purinergic receptors represent a potential therapeutic strategy for managing neuropathic pain [41,43,46].

Voltage-gated sodium channels, particularly the Nav1.7, Nav1.8, and Nav1.9 subtypes, play a pivotal role in the generation and propagation of action potentials in pain-sensing neurons [47,48]. Following nerve injury, there is an upregulation and altered distribution of sodium channels in both injured and neighboring intact neurons. This issue leads to enhanced excitability and ectopic firing, contributing to the development of neuropathic pain [47,48]. Thus, targeting specific sodium channel subtypes emerged as a potential strategy for alleviating neuropathic pain symptoms.

Voltage-gated calcium channels, including the N-type (Cav2.2), P/Q-type (Cav2.1), and T-type (Cav3) channels, are involved in neurotransmitter release and neuronal excitability [49,50]. Dysregulation of calcium channel activity is implicated in the development and maintenance of neuropathic pain. Increased calcium influx into nociceptive neurons can trigger the release of pro-inflammatory mediators, enhance excitatory synaptic transmission, and contribute to neuronal hyperexcitability [49,50]. Modulating specific calcium channel subtypes shows promise as a therapeutic approach for managing neuropathic pain.

Potassium channels play a crucial role in regulating neuronal excitability [51,52]. In neuropathic pain, there is a dysregulation of potassium channel function, leading to altered potassium ion dynamics and impaired hyperpolarization. This issue results in prolonged action potentials and the increased excitability of pain-sensing neurons. Targeting specific potassium channel subtypes may help restore the balance of neuronal excitability and provide therapeutic benefits for neuropathic pain management [51,52].

Acid-sensing ion channels (ASICs) are proton-gated ion channels expressed in sensory neurons and play a role in pain signaling [53,54]. Following nerve injury, changes in pH occur in the microenvironment surrounding the damaged nerves. Acidification can activate ASICs, leading to neuronal excitability, hyperalgesia, and the development of mechanical and thermal hypersensitivity [53,54]. Inhibiting or modulating ASIC activity shows potential in attenuating neuropathic pain symptoms.

Neuroinflammatory processes are involved in the development and progression of neuropathic pain [6,55]. Activated immune cells release pro-inflammatory molecules, such as cytokines, chemokines, and prostaglandins, which sensitize pain-sensing neurons, enhance pain transmission, and contribute to the maintenance of neuropathic pain [6,55]. There is growing scientific awareness of the fact that the main molecules responsible for the development of neuropathic pain are pro-inflammatory cytokines, especially interleukin-1b (IL-1b) [6]. These cytokines could initiate a series of neuroinflammation-related processes that can increase and intensify the initial injury, leading to the manifestation of chronic pain [55]. Furthermore, inflammation upregulates cyclo-oxygenase-2 (COX-2) activity, thus increasing the synthesis of prostaglandins (PGE) [7] and the release of pain-related neuropeptides [56]. Metalloproteinases (MMPs), which are primarily associated with tissue remodeling and inflammation in neurodegenerative disorders [57], also play crucial roles in nociception and hyperalgesia during the chronic phase of neuropathic pain [58,59].

Epigenetic modifications, including DNA methylation, histone modifications, and non-coding RNA expression, can influence gene expression patterns and alter pain sensitivity [60,61,62]. Epigenetic changes in key pain-related genes can contribute to the development and persistence of neuropathic pain [60,61,62].

These molecular mechanisms interact and influence each other, contributing to the complex nature of neuropathic pain. Targeting these mechanisms with specific medications or interventions holds promise for developing novel therapeutic approaches to alleviate neuropathic pain and improve the quality of life of affected individuals.

## 3. Current Pharmacotherapies in Neuropathic Pain

**Figure 2:**
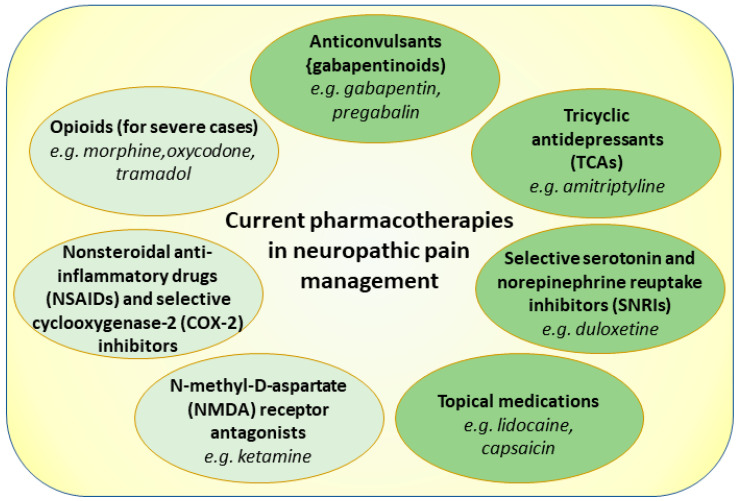
Present approach for treatment of neuropathic pain. First-line choices are shown in a darker color.

There are several popular and commonly used pharmacotherapies related to the management of neuropathic pain, including anticonvulsants (e.g., gabapentin and pregabalin); tricyclic antidepressants (TCAs) (e.g., amitriptyline); serotonin and norepinephrine re-uptake inhibitors (SNRIs) (e.g., duloxetine); topical agents (e.g., lidocaine patches or creams, capsaicin creams); non-steroidal anti-inflammatory drugs (NSAIDs) and selective cyclo-oxygenase-2 (COX-2) inhibitors, in the case of inflammation; N-methyl-D-aspartate (NMDA) receptor antagonists (e.g., ketamine), in specific cases, including central neuropathic pain and neuropathic pain in cancer; and opioids, such as morphine and oxycodone, which can be administered under thorough medical supervision for very severe cases [1] (Figure 2).

### 3.1. Gabapentinoids

Gabapentin are first-line treatments used to combat neuropathic pain [26,63]. The mechanism of action of gabapentinoids gabapentin and pregabalin is related to their ability to bind to the α2δ subunit of voltage-gated calcium channels, thus reducing the release of excitatory neurotransmitters [26,63].

Cao et al. performed the systematic review to compare the clinical efficacy of pregabalin and gabapentin in treating post-herpetic neuralgia and assessed the occurrence of adverse reactions [64]. In total, 14 randomized controlled trials involving 3545 patients were included in the study [64]. The findings showed that pregabalin was more effective than gabapentin in alleviating pain and improving global perception of pain and sleep [64]. However, gabapentin had a lower incidence of adverse events compared to pregabalin [64]. In the systematic review by Gimenez-Campos et al., the effectiveness of pregabalin and gabapentin in managing pain and disability caused by acute sciatica, as well as the associated adverse events, were assessed [65]. The review included 8 randomized controlled trials involving 747 participants [65]. The results showed that pregabalin and gabapentin were not effective in managing sciatica pain, as there were no statistically significant improvements in leg pain, low back pain, or functional disability compared to placebo or other treatments [65]. Shanthanna et al. performed the meta-analysis to assess the effectiveness and safety of gabapentinoids (pregabalin and gabapentin) in treating chronic low-back pain [66]. Eight randomized control trials were included in the study, and outcomes were guided by pain relief and safety as the primary measures. The study found that there was minimal improvement in pain relief when comparing gabapentin to placebo, as well as greater improvement in other analgesic groups when comparing pregabalin to other types of analgesic medication. Additionally, gabapentinoids were associated with adverse effects, such as dizziness, fatigue, difficulties with mentation, and visual disturbances, without any demonstrated benefit [66].

Gabapentin and pregabalin are effective in managing neuropathic pain associated with conditions like diabetic neuropathy and post-herpetic neuralgia [26,63]. The dosage of gabapentin is 900–3600 mg/day, while the dosage of pregabalin is 150–600 mg/day; the standard administration route is oral [67]. The side effects of gabapentinoids include central nervous system effects, e.g., dizziness, sedation, and cognitive impairment; gastrointestinal effects, like nausea, vomiting, and—rarely—peripheral edema; weight gain; fatigue; headache; dry mouth; visual disturbances; muscle pain; and mood changes [26,68].

Bao et al. assessed the analgesic efficacy of combining gabapentin and opioids for neuropathic cancer pain [69]. Seven relevant studies were included in the meta-analysis, which demonstrated that the combination of gabapentin and opioids effectively reduced pain intensity compared to opioids alone [69]. The pooled analysis showed a significant mean difference in pain intensity, supporting the use of gabapentin as an adjunctive therapy for neuropathic cancer pain [69].

Thus, gabapentinoids are effective in managing neuropathic pain, though their efficacy varies depending on the specific conditions. Due to side effects, individual response and tolerability should be considered when using these medications.

### 3.2. Tricyclic Antidepressants

Tricyclic antidepressants (TCAs) exert their analgesic effects in neuropathic pain through multiple mechanisms of action [70,71]. One of the main mechanisms involves the inhibition of the re-uptake of serotonin and noradrenaline in the pre-synaptic neurons, thereby increasing their availability in the synapsis [70,71]. This enhanced neurotransmitter activity modulates the pain signaling pathways, leading to a reduction in pain perception [70,71]. Moreover, additional mechanisms, such as N-methyl-D-aspartate receptor modulation and ion channel blockade, likely contribute to their pain-relieving effects [71].

Several studies investigated the effectiveness of TCA amitriptyline in various neuropathic pain conditions. Max et al. conducted a randomized controlled trial assessing the effectiveness of amitriptyline in post-herpetic neuralgia, revealing that it significantly decreased pain intensity and enhanced sleep quality compared to placebo [72]. Amitriptyline significantly reduced pain intensity and improved sleep and quality of life in patients with neuropathic pain of various etiologies [73]. In their systematic review and meta-analysis, Finnerup et al. determined that amitriptyline exhibited effectiveness in reducing pain intensity and improving sleep- and health-related quality of life among individuals with neuropathic pain [67].

The best dosage of amitriptyline for neuropathic pain can vary depending on multiple factors, including the individual’s age, medical condition, and response to treatment [74]. Typically, the initial dose of amitriptyline for neuropathic pain is low and gradually increased over time. The usual starting dose ranges from 10 to 25 mg, being taken orally at bedtime. The dosage may be increased by 10 to 25 mg per week until an effective dose is reached, which is often between 50 and 150 mg per day [74]. The most commonly encountered side effects of amitriptyline include weight gain, gastrointestinal symptoms like constipation, xerostomia, dizziness, headache, and somnolence [74].

In summary, tricyclic antidepressants (TCA), such as amitriptyline, are drugs with a moderate-to-high quality of evidence and a strong recommendation for use in the treatment of neuropathic pain [1,67].

### 3.3. Serotonin–Norepinephrine Re-Uptake Inhibitors

Serotonin–norepinephrine re-uptake inhibitors (SNRIs) are a class of medications that increase the levels of serotonin and norepinephrine by blocking their re-uptake, thus leading to reduced pain signals [75]. SNRIs are commonly used in the management of depression and anxiety disorders, though they are also effective in treating neuropathic pain [75]. SNRIs can be considered when tricyclic antidepressants and anticonvulsants are ineffective or contraindicated in neuropathic pain treatment [75]. Duloxetine is commonly used SNRI for neuropathic pain [75]. In randomized, placebo-controlled trials, the efficacy of duloxetine in diabetic peripheral neuropathic pain was assessed [76]. The study found that duloxetine at doses of 60 and 120 mg/day significantly reduced pain severity compared to placebo and improved overall functioning [76]. In study by Goldstein et al., patients with painful diabetic neuropathy were treated with duloxetine at doses of 60 or 120 mg/day, resulting in pain relief and increased overall quality of life compared to placebo [77]. Wernicke et al. performed a randomized, double-blind trial to evaluate the efficacy and safety of duloxetine in patients with chronic low-back pain and a neuropathic component [78]. Duloxetine at doses of 60 and 120 mg/day showed significant reductions in pain severity compared to placebo [78]. This systematic review found that duloxetine has a beneficial effect compared to placebo in the management of painful diabetic neuropathy [79]. In total, 23 studies were included, with 8 considered to be of high quality [79]. However, there was insufficient evidence to determine its superiority over pregabalin and amitriptyline, as only one trial was available for each comparison [79].

Duloxetine is typically prescribed for neuropathic pain at a starting dose of 30 mg once daily, which may be increased to 60 mg once daily based on individual response and tolerability [75]. Common side effects include nausea, dry mouth, constipation, dizziness, somnolence (excessive sleepiness), and sweating [75].

Like tricyclic antidepressants (TCAs), serotonin–norepinephrine re-uptake inhibitors, such as duloxetine, are drugs with a moderate-to-high quality of evidence and a strong recommendation for use in the treatment of neuropathic pain [1,67].

### 3.4. Lidocaine

Lidocaine is a widely used local anesthetic that gained attention as an alternative therapeutic option for neuropathic pain [80,81,82]. Lidocaine belongs to the class of amide-type local anesthetics and works by blocking sodium channels, thereby inhibiting the transmission of pain signals [80,81,82]. Lidocaine also modulates NMDA receptors, which play a role in chronic pain [80,81,82]. By inhibiting NMDA receptors, lidocaine interferes with pain-signal transmission and can reduce central sensitization [80,81,82]. This modulation contributes to lidocaine’s efficacy in treating conditions like neuropathic pain [80,81,82].

In addition to its local anesthetic properties, lidocaine demonstrated analgesic effects when administered systemically, making it suitable for the treatment of neuropathic pain in various clinical settings [80,81,82]. The use of lidocaine for neuropathic pain involves different formulations and routes of administration. Lidocaine patches or topical creams are commonly applied directly to the affected area, providing localized pain relief [83]. Additionally, lidocaine can be administered intravenously [84,85] or orally in the form of sustained-release formulations [86], allowing for systemic distribution and prolonged analgesic effects.

Numerous studies and clinical trials explored the efficacy and safety of lidocaine in neuropathic pain conditions, such as post-herpetic neuralgia, diabetic neuropathy, and neuropathic pain associated with spinal cord injury. These investigations focused on evaluating the analgesic efficacy, duration of pain relief, functional improvements, and overall patient satisfaction with lidocaine treatment. The analgesic efficacy of intravenous lidocaine (5 mg/kg i.v. over 30 min) was evaluated in patients with neuropathic pain in a randomized, controlled, and double-blind crossover trial, leading to a significant reduction in pain intensity compared to placebo, suggesting its potential as a treatment option for neuropathic pain [87]. However, in a single site randomized double-blind crossover trial, IV lidocaine infusion’s (5 mg/kg effectiveness was compared to active placebo infusion containing diphenhydramine (50 mg) in patients with chronic peripheral neuropathic pain, resulting in no significant long-term pain relief or improvement in quality of life [85]. Nevertheless, in a comprehensive literature review, the use of intravenous lidocaine infusion as a treatment for peripheral neuropathy was investigated, showing its effectiveness as a treatment option with minimal side effects [84]. Lidocaine can also be used in the form of sustained-release capsules, transmucosal patches, or oral gels/suspensions to exert longer lasting pain relief in cases of chronic or neuropathic pain [88,89,90,91,92].

The efficacy and safety of a topical 5% lidocaine medicated plaster was tested in patients with painful diabetic peripheral neuropathy in a randomized, double-blind, and placebo-controlled trial, providing significant pain relief compared to placebo, as well as a favorable safety profile [93]. In a literature review, topical lidocaine alone or in combination with other treatments, e.g., gabapentinoids, TCA, NSAIDs or, in severe cases, opioids, showed efficacy and safety for effective pain management in post-herpetic neuralgia, post-surgical pain, diabetic peripheral neuropathy, carpal tunnel syndrome, chronic lower back pain, and osteoarthritis [82].

Thus, lidocaine can provide significant pain relief in neuropathic pain conditions when administered intravenously. Topical lidocaine offers a localized analgesic option with minimal systemic adverse events. Lidocaine is generally safe when used in therapeutic doses for local anesthesia or pain management [94]. The toxic effects of lidocaine can occur in a dose-dependent manner. Excessive doses of lidocaine can lead to lidocaine toxicity, a condition that manifests through central nervous system (CNS) and cardiovascular symptoms [94]. Some possible side effects may include local skin reactions (with topical use), dizziness, drowsiness, headache, nausea, vomiting, metallic taste, and numbness or tingling at the site of application or infusion [94].

### 3.5. Capsaicin

Capsaicin is a naturally occurring compound found in chili peppers (*Capsicum annuum* L.) that is utilized for its analgesic properties in the management of neuropathic pain [95]. It is commonly available as a topical medication and is applied directly to the skin at the site of pain [96]. The mechanism of action of capsaicin involves the desensitization of nociceptive nerve fibers, which are responsible for transmitting pain signals to the brain. When capsaicin is applied topically, it binds to transient receptor potential vanilloid 1 (TRPV1) channels, causing a burning or stinging sensation, followed by a reduction in pain sensitivity due to the depletion of substance P, which is a neurotransmitter involved in the transmission of pain signals [96].

Capsaicin 8% dermal patches showed effectiveness comparable to centrally acting agents, like pregabalin, with potentially fewer systemic side effects, faster onset of action, and higher treatment satisfaction [97]. A review of 14 selected studies that reviewed the capsaicin 8% patch (Qutenza) witnessed significantly reduced average pain intensity in chronic post-surgical pain patients, showing only mild adverse effects [98].

The capsaicin 8% patch provided effective pain relief with reduced application frequency and minimal systemic side effects compared to oral medications, like gabapentinoids or TCAs, in patients with painful diabetic peripheral neuropathy [99]. In a randomized controlled trial, the application of the capsaicin 8% patch for neuropathic pain in individuals with spinal cord injury was investigated, showing significant pain reduction, with improvements observed for pain outcome and mobility, but not in quality of life, measures [100]. In a systematic review of 5 studies including 95 patients, the efficacy and tolerability of 8% capsaicin patch was evaluated for its effectivess in mitigating the effects of chemotherapy-induced peripheral neuropathy, providing significant pain relief [101].

Capsaicin is primarily used in the management of conditions such as post-herpetic neuralgia, diabetic neuropathy, and other forms of peripheral neuropathic pain [96]. It is considered a second-line treatment option when first-line treatments, such as oral medications, fail to provide adequate relief or are associated with significant side effects [102]. The concentration and frequency of application may vary depending on the specific product and the condition being treated [102]. While capsaicin is generally well-tolerated, mild and transient local skin reactions, such as redness, burning, or itching at the application site, could be observed [102]. Precautions should be taken to avoid contact with eyes, mucous membranes, and other sensitive areas [102].

### 3.6. Second Line Choices for Neuropathic Pain Treatment

When first-line treatments, such as antidepressants, anticonvulsants, and other medications, do not provide sufficient relief or are not well-tolerated, NMDA antagonists may be considered as an alternative option [74]. In neuropathic pain conditions, NMDA receptors can become overactive, leading to increased pain sensitivity and the development of central sensitization [103,104,105]. NMDA antagonists work through blocking the activity of NMDA receptors, thereby reducing the excitatory transmission of pain signals in the central nervous system [103,104,105]. By inhibiting NMDA receptor activation, these medications can help alleviate neuropathic pain and prevent the establishment of chronic pain states [103,104,105]. Ketamine is an anesthetic medication that, at lower doses, acts as a NMDA receptor antagonist [106]. It was shown to have analgesic properties, particularly in cases of refractory or severe neuropathic pain [106]. Ketamine can be administered intravenously, topically, or as an oral medication under careful medical supervision [106]. Other NMDA antagonists, such as memantine, dextromethorphan, and magnesium sulfate, were also studied for their potential efficacy in neuropathic pain management [107]. These medications work through different mechanisms to modulate NMDA receptor activity and reduce pain transmission [107]. Overall, NMDA antagonists show promise in managing neuropathic pain by targeting the underlying mechanisms of central sensitization [103,104,105]. However, their use for the management of neuropathic pain is generally reserved for cases where other treatments were ineffective due to several factors, including the potential for side effects, the need for careful dosing and monitoring, and the specialized administration requirements [103,104,105].

Nonsteroidal anti-inflammatory drugs (NSAIDs) are commonly used for pain relief and inflammation management when treating various conditions, including neuropathic pain [108,109]. While NSAIDs primarily work by inhibiting the activity of cyclo-oxygenase (COX) enzymes and reducing the production of inflammatory prostaglandins, their role in neuropathic pain management is somewhat limited [108,109]. Neuropathic pain is typically caused by damage or dysfunction of the nervous system, resulting in abnormal pain signaling [110,111]. Unlike inflammatory pain, neuropathic pain involves complex mechanisms that extend beyond inflammation and may not respond as effectively to NSAIDs. However, NSAIDs can still have some benefits in managing neuropathic pain in certain situations [74]. They may be helpful when neuropathic pain is accompanied by inflammation or there is an inflammatory component contributing to the pain. In such cases, NSAIDs can help to reduce inflammation and alleviate associated symptoms, leading to some pain relief.

Opioids, including morphine and oxycodone, are generally reserved for severe or refractory cases of neuropathic pain [103,112]. They may provide short-term relief, though their long-term efficacy in treating neuropathic pain is uncertain, and they can be associated with significant side effects and risks, including sedation, respiratory depression, constipation, nausea, itching, hormonal effects, and potential dependence and addiction [103,112]. Due to these considerations, opioids are typically prescribed at the lowest effective dose for the shortest duration possible [103,112]. They are often used as part of a comprehensive pain management plan that includes other non-opioid medications, physical therapy, behavioral interventions, and lifestyle modifications [103,112]. The traditional administration route of opioids for chronic pain management is oral, while the dosage varies depending on several factors, including the specific opioid being used, the severity of pain, the individual’s tolerance, and the presence of any underlying health conditions.

Tramadol and tapentadol are both centrally acting analgesics that are used for the management of moderate-to-severe pain, including neuropathic pain [74,113,114]. Tramadol acts as a weak mu-opioid receptor agonist and inhibits the re-uptake of norepinephrine and serotonin, and its analgesic effects are attributed to these combined actions [114,115]. Tapentadol, on the other hand, is a dual-action medication with mu-opioid receptor agonism and norepinephrine re-uptake inhibition [113]. It has a stronger affinity for the mu-opioid receptor compared to tramadol [113,115]. Tapentadol is considered to be more potent than tramadol, as it has a greater analgesic efficacy and a faster onset of action [113,115]. Both tramadol and tapentadol can cause similar side effects, such as nausea, dizziness, constipation, and drowsiness. However, tapentadol is generally associated with a lower incidence of gastrointestinal side effects than tramadol [113,115]. Tramadol has a lower potential for abuse compared to traditional opioids; however, it can still cause dependence and addiction in susceptible individuals [114]. Tapentadol has a lower risk of abuse compared to other opioids due to its dual mechanism of action and its reduced affinity for serotonin re-uptake inhibition, which is associated with the euphoric effects experienced via the use of some opioids [113,115].

Thus, the management of neuropathic pain requires a comprehensive approach that involves different treatment options. NMDA antagonists, such as ketamine and memantine, which target central sensitization mechanisms, are beneficial when inflammation accompanies neuropathic pain. Opioids, like morphine and oxycodone, are reserved for severe cases, but some carry risks [103,112]. Tramadol and tapentadol are centrally acting analgesics that are used for moderate-to-severe neuropathic pain, with tapentadol offering greater potency and fewer gastrointestinal side effects [115]. Each of these treatment choices has its advantages and considerations, and individual patient factors should guide the selection of second-line options for neuropathic pain management.

## 4. New Pharmacotherapies in Neuropathic Pain

**Figure 3:**
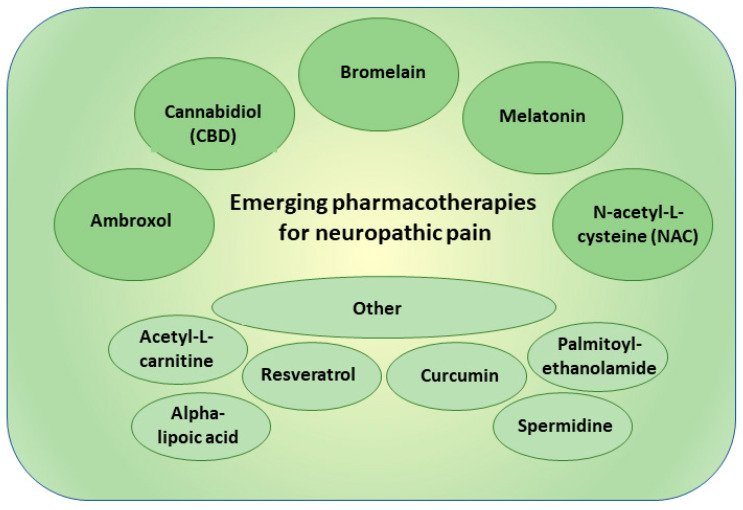
New approaches in neuropathic pain management. Main compounds are shown in a darker color.

Neuropathic pain, which is characterized by abnormal sensory processing due to nerve damage or dysfunction, often poses challenges in finding effective and well-tolerated therapies [110,111]. Traditional analgesics, such as opioids and NSAIDs, may provide limited relief or be associated with significant side effects [102]. The investigation into new drug targets and emerging pharmacotherapies in neuropathic pain could be of great interest in enhancing pain management and improving patient outcomes. In the context of neuropathic pain, repurposing drugs gained attention as a promising strategy for discovering novel treatment options [116]. Repurposing drugs for neuropathic pain offers several advantages in the drug development process. Firstly, utilizing drugs with established safety profiles and pharmacokinetic data allows for faster progress in pre-clinical and early clinical testing, thus saving time and resources. Secondly, the wide range of approved drugs across therapeutic classes provides numerous candidates for repurposing, increasing the likelihood of finding effective treatments. Thirdly, this approach opens the possibility of discovering novel targets and mechanisms for pain management, shedding light on previously unexplored pathways. Lastly, repurposed drugs can be translated to clinical use more quickly, benefiting patients in urgent need of effective neuropathic pain treatments [116]. The main new pharmacotherapies for neuropathic pain are summarized in Figure 3.

### 4.1. Ambroxol

Ambroxol, which is an active metabolite of bromhexine, was safely utilized for many years in the management of acute respiratory conditions, like bronchitis and chronic respiratory diseases, as it acts as an expectorant and mucolytic agent [117,118]. Furthermore, ambroxol recently showed potential in the management of neuropathic pain due to its multiple mechanisms of action [119]. Ambroxol modulates the activity of voltage-gated sodium channels, specifically Nav1.8, which are involved in the generation and propagation of pain signals [120]. By inhibiting Nav1.8 channels, ambroxol may reduce the excitability of nociceptive neurons and dampen neuropathic pain transmission [120]. Several pre-clinical studies investigated the analgesic properties of ambroxol in various animal models of neuropathic pain. In animal models of chronic, neuropathic, and inflammatory pain, ambroxol was tested using the formalin paw model and two mononeuropathy models, as well as a monoarthritis model in rats [121]. At a dosage of 1 g/kg, which is equivalent to clinical use, ambroxol effectively reduced pain symptoms and even reversed pain behavior. Its efficacy surpassed that of gabapentin (at 100 mg/kg), suggesting that a Nav1.8-preferring Na^+^ channel blocker, like ambroxol, can suppress chronic, neuropathic, and inflammatory pain at clinically achievable plasma levels [121]. The effectiveness of pregabalin and ambroxol, either alone or in combination, in alleviating oxaliplatin-induced cold allodynia was evaluated using the mouse cold plate test [122]. The combination of ambroxol and pregabalin demonstrated an antiallodynic effect, whereas ambroxol preferentially bound to mouse Na(v)1.6 and Na(v)1.9 channels [122]. Additionally, ambroxol demonstrated efficacy in alleviating neuropathic spinal cord injury pain in rats by reducing hypersensitivity below the injury level, possibly through inhibiting peripheral sodium channels [123]. Thus, in vivo data suggest that ambroxol might be useful as a therapeutic alternative for the treatment of neuropathic pain.

While there is limited clinical data available on the alternative uses of ambroxol, some studies explored its analgesic effects. Topical ambroxol cream (20%) was used for the treatment of severe neuropathic pain in seven patients unresponsive to standard therapies, e.g., lidocaine or capsaicin patches, in the retrospective study [124], providing individual pain reductions within a period lasting for several hours. The cream effectively reduced pain attacks and was well tolerated without any reported side effects or skin changes [124]. In a study involving eight patients with complex regional pain syndrome symptoms lasting for less than 12 months, topical 20% ambroxol cream was used in addition to standard therapy, i.e., lidocaine or capsaicin patches [125]. The results showed a reduction in spontaneous pain, pain on movement, edema, allodynia, hyperalgesia, and skin reddening, as well as improvement in motor dysfunction and skin temperature [125]. In a study involving patients with trigeminal neuralgia, topical ambroxol 20% cream was used in addition to standard treatment [126]. All patients experienced pain reduction, with attacks being reduced and pain intensity decreasing; the pain relief was observed within 15–30 min and lasted for 4–6 h [126]. No side effects or skin changes were reported, and oral medication was reduced in some cases [126].

Ambroxol is generally considered safe and well tolerated when used within the recommended dosage range [118]. Common side effects may include gastrointestinal disturbances, such as nausea and vomiting, though side effects are typically mild and transient [118]. The use of ambroxol in neuropathic pain management is an emerging area of research; therefore, further clinical studies are required to evaluate its efficacy, optimal dosing regimens, and long-term safety profile, as well as the effects of combining ambroxol with other analgesic agents [120].

### 4.2. Cannabidiol

Cannabidiol (CBD) is a naturally occurring non-psychoactive cannabinoid compound that is found in the cannabis plant (*Cannabis sativa* L.). CBD was previously explored for various medical conditions and gained significant attention in recent years for its potential analgesic [127,128], anti-inflammatory [129,130,131], neuroprotective [132], anticonvulsant [129], antiemetic [133], and spasmolytic [134] properties.

CBD emerged as a prospective candidate for the treatment of neuropathic pain due to its potential analgesic and anti-inflammatory effects [127,128,129,130,131]. CBD interacts with the endocannabinoid system (ECS) in the body, which plays a role in regulating various physiological processes, including pain perception [18,135,136]. CBD acts on cannabinoid receptors, particularly the CB1 and CB2 receptors, to modulate pain signaling and reduce inflammation [18,135,136]. The G protein-coupled receptors CB1 and CB2, which belong to the cannabinoid receptor family, play a crucial role in regulating various intracellular signaling pathways [18]. These pathways involve the activation of mitogen-activated protein kinases (MAPK), phosphorylation, and the modulation of potassium and calcium channels [18]. CB1 receptor activation leads to a decrease in neuronal excitability and the release of neurotransmitters, such as gamma-aminobutyric acid and glutamate, in regions of the brain involved in nociception [19]. On the other hand, CB2 receptors are primarily found in immune tissues (e.g., spleen and tonsils) and immune cells (e.g., monocytes, B and T cells), with some presence in the brain. Activation of peripheral CB2 receptors produces anti-inflammatory and immunomodulatory effects, contributing to the alleviation of inflammatory and neuropathic pain [20,21].

**Figure 4:**
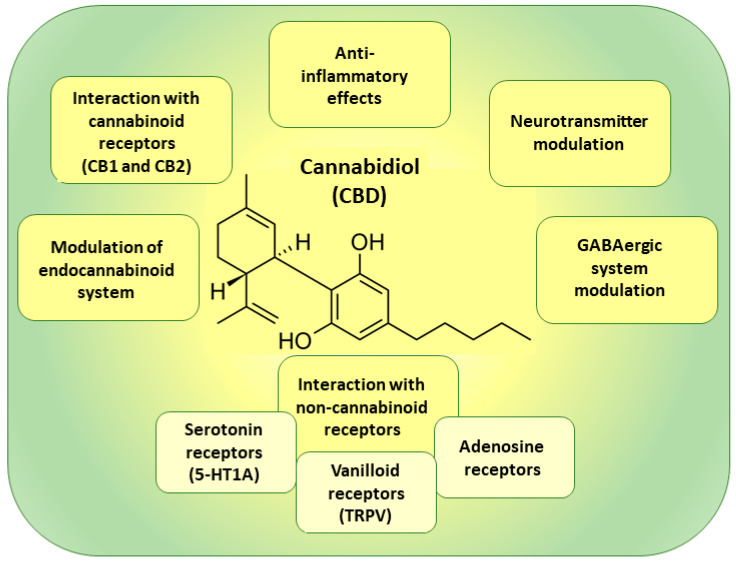
Mechanisms of action of cannabidiol (CBD) in pain relief.

CBD could also interact with other receptors and ion channels involved in pain transmission, such as transient receptor potential (TRP) channels [18,137,138,139]. CBD mechanisms of action involved in the treatment of neuropathic pain are summarized in Figure 4.

Multiple pre-clinical and clinical studies demonstrated CBD’s potential to alleviate neuropathic pain symptoms [140,141]. CBD could reduce pain, improve sleep quality, and enhance overall quality of life in individuals with multiple sclerosis (MS), diabetic neuropathy, and post-herpetic neuralgia [83,140].

In in vivo studies, the antinociceptive effect of cannabidiol (CBD) (from 2.5 to 20 mg/kg i.p.) as an acute treatment for neuropathic pain induced by spinal cord injury was investigated in female Wistar rats [142]. The results demonstrated a dose-dependent reduction in nociceptive behaviors, decreased lipid peroxidation levels, and increased GSH concentration, indicating the antioxidant effects of CBD [142]. The effects of cannabidiol (CBD) on neuropathic pain induced by paclitaxel were investigated using male C57BL6 mice [143]. CBD treatment effectively prevented paclitaxel-induced neuropathic pain and was associated with inhibition of type 4 Toll-like receptors (TLR4) and microglia activation [143]. CBD also increased the levels of endocannabinoids and reduced pro-inflammatory cytokine levels in the spinal cord [143]. The findings suggest that CBD’s effects on neuropathic pain may involve modulation of the TLR4 pathway and activation of the endocannabinoid system [143]. CBD and β-caryophyllene, which are two cannabis constituents, when acting individually and in combination, showed analgesic effects in a rat model of chronic spinal cord injury pain [144]. The combination produced enhanced pain reduction with minimal side effects, implying that the co-administration of CBD and β-caryophyllene could offer a promising treatment option for chronic spinal cord injury pain [144]. The interaction between these compounds involved CB1 receptors, highlighting a novel mechanism of action [144].

In comprehensive literature review, 30 randomized controlled trials and other studies were analyzed, revealing the promising effects of cannabis in refractory multiple sclerosis, cancer pain (especially in advanced stages), and chronic rheumatic pain [145]. Cannabis-based medications were found to be more effective than herbal strains containing tetrahydrocannabinol, though further research is required to fully understand their benefits and risks [145]. In another review, the effectiveness of cannabis-based medications for chronic neuropathic pain was assessed [146]. In total, 17 randomized placebo-controlled trials were analyzed, which involved 861 patients with neuropathic pain. Meta-analysis revealed that THC/CBD, THC, and dronabinol significantly reduced pain intensity compared to placebo [146]. Patients taking THC/CBD were more likely to achieve a 30% reduction in pain [146]. The review of 25 randomized controlled trials involving adults with multiple sclerosis (MS) revealed that nabiximols, i.e., a combination of THC and CBD, likely reduced muscle-tightening severity in the short term; however, the effects of cannabinoids on chronic neuropathic pain and quality of life were uncertain [147]. Cannabinoids were associated with a slight increase in treatment discontinuation and the risk of nervous system and psychiatric disorders [147]. In the double-blind, placebo-controlled study, the analgesic effects of acute CBD were examined in healthy non-cannabis users [148]. The study found that CBD did not consistently improve pain threshold or tolerance [148]. CBD also had modest dose-dependent effects on mood and subjective drug effects related to abuse liability; however, oral CBD was deemed safe and well tolerated, with minor decreases in blood pressure [148].

CBD is usually administered orally at a dosage range of 2–25 mg/kg/day, depending on the individual patient’s response and tolerability. CBD is well tolerated and has relatively few serious adverse effects [149]; however, drug–drug interactions, diarrhea, fatigue, vomiting, somnolence and hepatic abnormalities were reported in several studies [150,151]. Due to adverse reactions, cannabinoid therapy should not be used for the patients with severe psychiatric, cardiac, renal, or hepatic disorders [152,153]

Despite CBD’s potential for neuropathic pain management, additional research is necessary to better understand its mechanisms of action, optimal dosage, long-term safety, and possible drug–drug interactions. Additionally, regulatory frameworks that regulate the use of CBD can vary between countries and regions; therefore, it is important to be aware of the legal considerations.

### 4.3. Bromelain

Bromelain is an enzyme derived from the pineapple plant (*Ananas comosus* L. Merr.) and is primarily known for its therapeutic applications in the field of digestive health. Bromelain is commonly recognized for its proteolytic properties. It contains a mixture of enzymes, including proteases; therefore, it is widely used as a digestive aid, particularly to improve protein digestion and reduce digestive discomfort, especially in individuals experiencing pancreatic insufficiency or other digestive disorders. Bromelain is a safe-to-use nutraceutical that lacks side effects.

While the main application of bromelain is related to digestion, there is limited scientific evidence supporting its direct use for neuropathic pain management. Bromelain’s potential anti-inflammatory properties and ability to modulate certain biological processes led to discussion about its potential use in neuropathic pain management.

In a rat model of neuropathic pain induced via sciatic nerve ligation, treatment with bromelain resulted in significant reductions in thermal hyperalgesia and mechanical allodynia [154]. It also facilitated the recovery of sciatic function and structural integrity [154]. Additionally, bromelain administration in another rat model of neuropathic pain showed a decrease in characteristic signs of neuropathic pain [155].

Bromelain was found to alleviate neuropathic pain and anxiety-like behaviors in a rat model of peripheral neuropathy [156]. It reduced pro-inflammatory cytokines, nitrate levels, and iNOS expression in the sciatic nerve, suggesting that bromelain’s antinociceptive and anxiolytic effects are linked to its ability to reduce inflammation [156].

The efficacy and safety of OPERA^®^, which is a dietary supplement containing α-lipoic acid, Boswellia Serrata, methylsulfonylmethane, and bromelain, was evaluated in patients with chemotherapy-induced peripheral neuropathy (CIPN) [157]. In total, 25 patients with CIPN were enrolled, and their neuropathy symptoms were evaluated over a 12-week period. The primary endpoint was the change in measured scores after 12 weeks of OPERA^®^ therapy compared to the baseline. Secondary endpoints included the reduction in neuropathy symptoms after 12 weeks of treatment. The results showed a reduction in pain perceived by patients and improvement in sensor and motor neuropathic impairment. The OPERA^®^ supplement was well tolerated, with no significant increase in toxicity or interactions with other therapies. Further research, including randomized controlled trials, is needed to confirm its potential benefits in a larger patient population [157]. Bromelain is administered orally, while the ideal dosage is not yet established and may vary depending on the specific product and its concentration, as well as the severity of neuropathic pain and the individual’s response to treatment. In animal studies, dosages of 30–50 mg/kg per os were used [154].

Bromelain may help to reduce pain and inflammation by inhibiting inflammatory mediators, promoting tissue healing, and modulating immune responses. However, more research is needed to establish the efficacy and safety of bromelain specifically for neuropathic pain.

### 4.4. Melatonin

The endogenous hormone melatonin, also known as N-acetyl-5-methoxytryptamine, is primarily synthesized from the amino acid tryptophan. Tryptophan is converted into 5-hydroxytryptophan (5-HTP) by the enzyme tryptophan hydroxylase. Next, 5-HTP is further transformed into serotonin (5-hydroxytryptamine) by the enzyme aromatic L-amino acid decarboxylase. Serotonin serves as the precursor to melatonin synthesis. In the pineal gland, serotonin is converted into N-acetyl serotonin by the enzyme serotonin N-acetyltransferase, and is then methylated by the enzyme acetyl serotonin O-methyltransferase to form melatonin. However, it can also be produced in various organs and cells, including the brain, bone marrow, retina, skin, lens, and lymphocytes [158,159]. In adults, a constant secretion of approximately 30 µg/day of melatonin occurs, though its synthesis increases in the evening, reaching a peak concentration in the middle of the dark period [158,159]. Melatonin plays a crucial role in the regulation of circadian rhythms [158,159] and exhibits antioxidant properties, protecting against lipid peroxidation, inflammation, and tumor growth and promoting apoptosis and mitochondrial function [159,160]. Aging is associated with a decline in melatonin synthesis, leading to conditions such as insomnia, particularly in cases of Alzheimer’s disease; cardiovascular disorders; and cancer [161].

The cellular effects of melatonin are mediated through interactions with specific receptors and intracellular targets, including transporters, ion binding proteins, enzymes, cytoskeletal components, and mitochondria [162,163,164,165]. Melatonin is capable of freely crossing cell membranes and the blood–brain barrier, allowing it to exert its actions in various tissues and organs [166]. These interactions enable melatonin to modulate the diverse cellular processes and signaling pathways involved in its beneficial effects.

**Figure 5:**
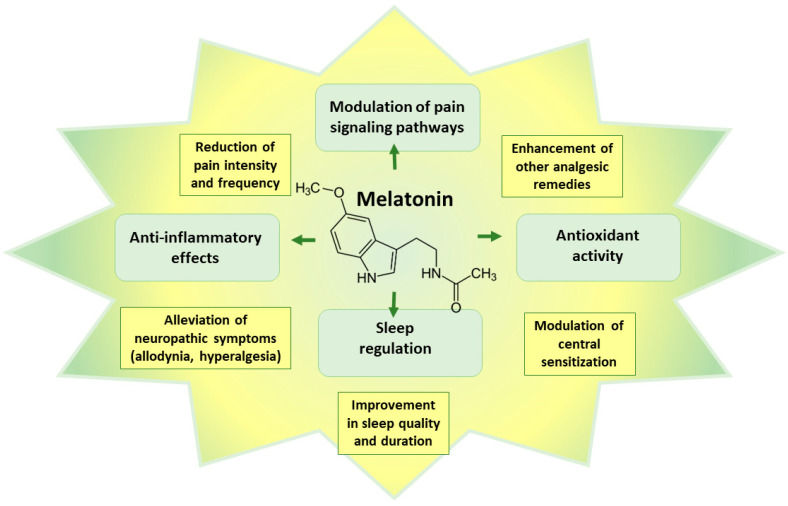
Main therapeutic effects of melatonin in neuropathic pain.

Melatonin exhibits various mechanisms of action that contribute to its potential therapeutic effects in neuropathic pain [167,168], which are summarized in Figure 5.

Firstly, it can modulate pain signaling pathways through interaction with receptors involved in pain regulation, such as opioid, adrenergic, and cannabinoid receptors [169,170]. The effects of melatonin also result from activation of MT1 and MT2 melatonin receptors, which leads to reduced cyclic AMP formation and reduced nociception [171]. Through these interactions, melatonin can effectively modulate pain perception and reduce pain transmission [171]. Secondly, melatonin possesses anti-inflammatory properties, suppressing the production of pro-inflammatory cytokines and molecules, like IL-1β, TNF-α, and NOS [171], which are associated with the inflammatory response observed in neuropathic pain. Additionally, melatonin acts as a powerful antioxidant, protecting cells from oxidative stress and minimizing neuronal damage and inflammation [171]. Melatonin is generally considered safe and non-toxic, with only mild side effects, such as dizziness, headache, nausea, and sleepiness, reported even at high doses [172].

In the context of neuropathic pain, melatonin demonstrated therapeutic effects in clinical and pre-clinical studies [167,173,174]. It could effectively reduce pain intensity and frequency; improve sleep quality and duration; alleviate neuropathic symptoms, like allodynia and hyperalgesia; and modulate central sensitization, which is a key mechanism underlying neuropathic pain [168,174]. Furthermore, when used in combination with conventional analgesic medications, melatonin showed the potential to enhance their efficacy [167].

The effects of melatonin in a mononeuropathy pain model on Sprague–Dawley rats were assessed in an in vivo study [175]. Administration of melatonin (5–10 mg/kg) on the 14th day after surgery reduced thermal hyperalgesia and modulated the nitroxidergic system in the dorsal root ganglia and skin [175]. Melatonin (37.5, 75, or 150 mg/kg once per day p.o. 30 min before lysophosphatidylcholine treatment for 3 days) also reduced neuropathic pain, behavior, and glial activation through MT2 melatonin receptor modulation in a rat model of lysophosphatidylcholine-induced demyelination neuropathy [176]. Intrathecal administration of melatonin ameliorated the neuroinflammation- mediated sensory and motor dysfunction in a rat model with compression spinal cord injury [177]. Exogenous melatonin (10 mg/kg) alleviated neuropathic pain-induced affective disorders in rats by suppressing NF-κB/ NLRP3 pathway and apoptosis [178].

While preliminary studies suggested potential benefits of melatonin in neuropathic pain, it is important to note that further research is necessary to fully comprehend the precise mechanisms of action of melatonin and determine the optimal approach for its application as a pain reliever.

### 4.5. N-acetyl-L-cysteine

N-acetyl-L-cysteine (NAC) is a modified form of the amino acid cysteine. It is primarily recognized for its role as an antidote in cases of acetaminophen overdose [179,180]. It helps to replenish cellular levels of glutathione, which is a crucial antioxidant that protects the liver from the toxic effects of acetaminophen metabolites [179,180]. Additionally, NAC is used as a mucolytic agent to help break down and thin mucus in respiratory conditions, such as chronic bronchitis, cystic fibrosis, and chronic obstructive pulmonary disease [179,180].

N-acetyl-L-cysteine (NAC) was studied for its possible therapeutic effects in neuropathic pain in recent years [179,180]. The antioxidant and anti-inflammatory effects of NAC are hypothesized to play a role in its analgesic effects [179,180]. Oxidative stress and inflammation are known to contribute to nerve damage and the development of neuropathic pain. NAC, as a precursor of glutathione, can enhance the body’s antioxidant defenses and help to reduce oxidative stress [180,181]. Moreover, it may modulate inflammatory responses and inhibit the release of pro-inflammatory molecules [182]. NAC could act as a neuroprotective agent by modulating the activity of various neurotransmitters and receptors involved in pain transmission [183,184]. It interacts with glutamatergic and GABAergic systems, influencing excitatory and inhibitory signaling in the central nervous system [183,184]. NAC can regulate the release and re-uptake of neurotransmitters, including glutamate, which plays a crucial role in neuropathic pain [183]. Additionally, NAC was found to modulate the activity of ion channels, such as voltage-gated sodium channels, which are involved in pain signaling [184].

NAC modulated Ca^2+^ influx through a TRPM2 channel in intracellular GSH-depleted rat dorsal root ganglions [184] or in the diabetic rat dorsal root ganglions in vitro [185]. NAC (100 mg/kg, i.p.) caused analgesia by reinforcing the endogenous activation of type-2 metabotropic glutamate receptors in mice in vivo [183]. Moreover, NAC (100 mg/kg, i.p., either single injection or daily injections for seven days) induced analgesia in a mouse model of painful diabetic neuropathy [186]. NAC (100 mg/kg/day, i.p. for 3 or 10 days) had no effect on the spinal cord glutathione system, but reduced nitric-oxide metabolites in rats with neuropathic pain [182]. Both the in vitro (0.1 mM) and in vivo (50, 100, and 200 mg/kg p.o.) applications of NAC significantly suppressed the activity of matrix metalloproteinases, thus alleviating the neuropathic pain in the chronic constrictive injury model in rats [187]. Furthermore, NAC (150 mg/kg/day i.p. for 1, 3, or 7 days) decreased spinal cord oxidative stress biomarkers in rats with neuropathic pain [181]. In the study on the role of astrocyte–neuron interactions in diabetic neuropathic pain, increased expression of chemokine CXC receptor 4 (CXCR4) and connexin 43 (CX43) were observed in the spinal cord dorsal horn of rats with diabetic neuropathic pain, whereas the CXCR4 antagonist AMD3100 and the antioxidant NAC reversed nociceptive behavior [188].

Heidari et al. investigated the effects of oral N-acetylcysteine (NAC) as an adjunct therapy for painful diabetic neuropathy (PDN) [189]. A total of 113 patients with type 2 diabetes and PDN were randomly assigned to receive pregabalin and placebo or pregabalin and NAC for 8 weeks (pregabalin at a dose of 150 mg per day, compared to NAC and matched placebo at doses of 600 mg twice a day). Patients receiving pregabalin and NAC showed greater reductions in pain scores and sleep interference compared to those receiving pregabalin and placebo. More responders and improvements in global impression of change were observed in the pregabalin and NAC group. NAC also reduced oxidative stress biomarkers and increased antioxidant levels [189]. The systematic review was performed to evaluate the efficacy and safety of NAC in the treatment of chronic pain [190]. Nine studies involving different chronic pain conditions were included. The pooled analysis of three randomized controlled trials did not show a significant reduction in pain intensity or improvement in functional outcomes or quality of life with NAC. However, sensitivity analysis suggested a potential effect on pain intensity and function [190].

While pre-clinical studies and some clinical trials showed promising results regarding the analgesic effects of NAC in neuropathic pain [180], further research is needed to establish its efficacy, optimal dosing, and long-term safety profile. Furthermore, the mechanisms through which NAC exerts its analgesic effects in neuropathic pain require additional investigation.

It is important to note that NAC is generally considered safe when used within recommended dosages (from 600 mg to 2400 mg per day) [180]. However, it may cause side effects, such as gastrointestinal symptoms (nausea, vomiting, diarrhea), allergic reactions, and potential interactions with certain medications [180].

### 4.6. Other Experimental Therapies

There are several non-traditional compounds that show potential for the management of neuropathic pain [110,111,191] (Figure 2). Acetyl-L-carnitine was investigated for its potential role in managing neuropathic pain [192,193]. It exerts its effects through multiple mechanisms, including modulation of neurotransmitters such as glutamate and GABA; promotion of nerve regeneration, antioxidant activity, and anti-inflammatory effects; and modulation of synaptic plasticity [193,194,195]. By influencing these processes, acetyl-L-carnitine may help to regulate pain signaling, repair damaged nerves, reduce oxidative stress and inflammation, and modulate abnormal neuronal activity associated with neuropathic pain [193,195]. Alpha–lipoic acid is an antioxidant that was previously studied for its neuroprotective and analgesic effects in suppressing neuropathic pain [196]. It is supposed to reduce oxidative stress and inflammation, thereby alleviating pain symptoms [196]. Palmitoylethanolamide is an endogenous fatty acid that acts as a modulator of inflammation and pain [197]. It was previously shown to exert analgesic effects by targeting various pathways involved in neuropathic pain, including the activation of cannabinoid receptors and the inhibition of inflammatory mediators [197]. Spermidine is a naturally occurring polyamine that plays essential roles in various cellular processes, including cell growth, differentiation, and neuronal function [198]. Studies indicate that spermidine may alleviate pain hypersensitivity, modulate neurotransmitter systems, and promote neuroprotection [198]. With its favorable safety profile, spermidine supplementation could offer a viable option for managing neuropathic pain, although further research is needed to determine its mechanisms of action and optimal usage in human subjects [198]. Resveratrol is a natural compound found in grapes, berries, and other plants [199,200]. Resveratrol demonstrated anti-inflammatory and analgesic properties in pre-clinical studies of neuropathic pain, modulating multiple signaling pathways associated with pain and inflammation [199,200]. Curcumin, which is a polyphenolic compound derived from turmeric [201,202], was previously investigated for its potential in neuropathic pain management due to its anti-inflammatory and anti-oxidant properties. Curcumin may modulate pain signaling pathways and inhibit the production of pro-inflammatory molecules [201,202]. While further research is needed to establish their efficacy and safety, these compounds hold promise as alternative approaches for alleviating neuropathic pain and improving the quality of life for individuals suffering from this challenging condition.

Non-coding RNA molecules play a significant role in the development and regulation of neuropathic pain [203]. These RNA molecules, including microRNAs (miRNAs) and long non-coding RNAs (lncRNAs), were found to be involved in various aspects of neuropathic pain, such as neuronal plasticity, inflammation, and immune responses [203]. MiRNAs are small RNA molecules that regulate gene expression by binding to messenger RNAs (mRNAs) and inhibiting their translation or promoting their degradation [203]. In neuropathic pain, specific miRNAs were identified as key regulators of pain-related pathways. They can modulate the expression of genes involved in neuronal sensitization, synaptic plasticity, and inflammatory responses. By targeting these genes, miRNAs can influence the development and maintenance of neuropathic pain [203]. LncRNAs, on the other hand, are longer RNA molecules that do not encode proteins, but have important regulatory functions in cellular processes. Several lncRNAs are implicated in neuropathic pain by influencing gene expression, chromatin remodeling, and epigenetic modifications [203]. They can act as scaffolds, decoys, or guides to interact with proteins and other regulatory molecules, ultimately affecting the expression of pain-related genes [203]. Research into non-coding RNAs in neuropathic pain is still ongoing, and the specific mechanisms through which they contribute to pain pathology are being elucidated [203]. Understanding their roles may lead to the development of novel diagnostic markers and therapeutic targets for neuropathic pain management [203].

## 5. Further Perspectives in Neuropathic Pain Management

The management of neuropathic pain requires a multi-faceted approach, and several non-traditional compounds show promise in providing relief [110,111]. Natural remedies, such as capsaicin, alpha-lipoic acid, and botanical extracts, demonstrate analgesic effects and have the potential to alleviate neuropathic pain symptoms [111]. Additionally, neurotrophic factors like nerve growth factor (NGF) showed promising results in pre-clinical studies [204]. Moreover, complementary therapies, such as acupuncture and mind–body interventions, may offer alternative strategies for pain relief [28].

Although non-traditional compounds show promise in relieving neuropathic pain, additional research is required to evaluate their efficacy, safety, and appropriate dosage protocols. Comprehensive long-term studies are necessary to investigate the sustained therapeutic effects and the potential for disease progression prevention. Furthermore, conducting further clinical trials will enable the comparative assessment of various non-traditional compounds and combination therapies in terms of their effectiveness. An improved understanding of the underlying mechanisms of action associated with these compounds will also facilitate the development of targeted treatment approaches.

Furthermore, there is a need for studies investigating the potential synergistic effects of non-traditional compounds with conventional medications used for neuropathic pain. Combination therapies may enhance analgesic outcomes and reduce reliance on high doses of single agents, thereby minimizing side effects. Additionally, research focusing on personalized medicine approaches, which consider individual patient characteristics, including different and mixed pain mechanisms and sensory and genetic profiles, may help identify subgroups of patients who are more likely to benefit from specific non-traditional remedies.

## References

[1] Finnerup N.B., Kuner R., Jensen T.S. (2021). Neuropathic Pain: From Mechanisms to Treatment. Physiol. Rev..

[2] Fitzcharles M.A., Cohen S.P., Clauw D.J., Littlejohn G., Usui C., Häuser W. (2021). Nociplastic pain: Towards an understanding of prevalent pain conditions. Lancet.

[3] Jayakar S., Shim J., Jo S., Bean B.P., Singeç I., Woolf C.J. (2021). Developing nociceptor-selective treatments for acute and chronic pain. Sci. Transl. Med..

[4] Treede R.D., Jensen T.S., Campbell J.N., Cruccu G., Dostrovsky J.O., Griffin J.W., Hansson P., Hughes R., Nurmikko T., Serra J. (2008). Neuropathic pain: Redefinition and a grading system for clinical and research purposes. Neurology.

[5] International Association for the Study of Pain (IASP) (2023). IASP Terminology. https://www.iasp-pain.org/terminology#Nociplasticpain.

[6] Sacerdote P., Franchi S., Moretti S., Castelli M., Procacci P., Magnaghi V., Panerai A.E. (2013). Cytokine modulation is necessary for efficacious treatment of experimental neuropathic pain. J. Neuroimmune Pharmacol..

[7] Gao Y.J., Ji R.R. (2010). Targeting astrocyte signaling for chronic pain. Neurotherapeutics.

[8] Du Z., Zhang J., Han X., Yu W., Gu X. (2023). Potential novel therapeutic strategies for neuropathic pain. Front. Mol. Neurosci..

[9] Soliman N., Kersebaum D., Lawn T., Sachau J., Sendel M., Vollert J. (2023). Improving neuropathic pain treatment—By rigorous stratification from bench to bedside. J. Neurochem..

[10] Truini A. (2017). A Review of Neuropathic Pain: From Diagnostic Tests to Mechanisms. Pain Ther..

[11] Yin Q., Zou T., Sun S., Yang D. (2023). Cell therapy for neuropathic pain. Front. Mol. Neurosci..

[12] Guimarães Pereira J.E., Ferreira Gomes Pereira L., Mercante Linhares R., Darcy Alves Bersot C., Aslanidis T., Ashmawi H.A. (2022). Efficacy and Safety of Ketamine in the Treatment of Neuropathic Pain: A Systematic Review and Meta-Analysis of Randomized Controlled Trials. J. Pain Res..

[13] Kamp J., Van Velzen M., Olofsen E., Boon M., Dahan A., Niesters M. (2019). Pharmacokinetic and pharmacodynamic considerations for NMDA-receptor antagonist ketamine in the treatment of chronic neuropathic pain: An update of the most recent literature. Expert Opin. Drug Metab. Toxicol..

[14] Coluzzi F., Rullo L., Scerpa M.S., Losapio L.M., Rocco M., Billeci D., Candeletti S., Romualdi P. (2022). Current and Future Therapeutic Options in Pain Management: Multi-mechanistic Opioids Involving Both MOR and NOP Receptor Activation. CNS Drugs.

[15] Gamble M.C., Williams B.R., Singh N., Posa L., Freyberg Z., Logan R.W., Puig S. (2022). Mu-opioid receptor and receptor tyrosine kinase crosstalk: Implications in mechanisms of opioid tolerance, reduced analgesia to neuropathic pain, dependence, and reward. Front. Syst. Neurosci..

[16] Li L., Chen J., Li Y.Q. (2023). The Downregulation of Opioid Receptors and Neuropathic Pain. Int. J. Mol. Sci..

[17] Vincenzi M., Milella M.S., D’Ottavio G., Caprioli D., Reverte I., Maftei D. (2022). Targeting Chemokines and Chemokine GPCRs to Enhance Strong Opioid Efficacy in Neuropathic Pain. Life.

[18] Almogi-Hazan O., Or R. (2020). Cannabis, the Endocannabinoid System and Immunity-the Journey from the Bedside to the Bench and Back. Int. J. Mol. Sci..

[19] Laprairie R.B., Bagher A.M., Kelly M.E., Denovan-Wright E.M. (2015). Cannabidiol is a negative allosteric modulator of the cannabinoid CB1 receptor. Br. J. Pharmacol..

[20] Onaivi E.S., Ishiguro H., Gong J.P., Patel S., Perchuk A., Meozzi P.A., Myers L., Mora Z., Tagliaferro P., Gardner E. (2006). Discovery of the presence and functional expression of cannabinoid CB2 receptors in brain. Ann. N. Y. Acad. Sci..

[21] Van Sickle M.D., Duncan M., Kingsley P.J., Mouihate A., Urbani P., Mackie K., Stella N., Makriyannis A., Piomelli D., Davison J.S. (2005). Identification and functional characterization of brainstem cannabinoid CB2 receptors. Science.

[22] Carrasco C., Naziroǧlu M., Rodríguez A.B., Pariente J.A. (2018). Neuropathic Pain: Delving into the Oxidative Origin and the Possible Implication of Transient Receptor Potential Channels. Front. Physiol..

[23] De Logu F., Geppetti P. (2019). Ion Channel Pharmacology for Pain Modulation. Handb. Exp. Pharmacol..

[24] Iannone L.F., De Logu F., Geppetti P., De Cesaris F. (2022). The role of TRP ion channels in migraine and headache. Neurosci. Lett..

[25] Lee K., Jo Y.Y., Chung G., Jung J.H., Kim Y.H., Park C.K. (2021). Functional Importance of Transient Receptor Potential (TRP) Channels in Neurological Disorders. Front. Cell Dev. Biol..

[26] McAnally H., Bonnet U., Kaye A.D. (2020). Gabapentinoid Benefit and Risk Stratification: Mechanisms Over Myth. Pain Ther..

[27] Petroianu G.A., Aloum L., Adem A. (2023). Neuropathic pain: Mechanisms and therapeutic strategies. Front. Cell Dev. Biol..

[28] Macone A., Otis J.A.D. (2018). Neuropathic Pain. Semin. Neurol..

[29] Casey A.B., Cui M., Booth R.G., Canal C.E. (2022). “Selective” serotonin 5-HT(2A) receptor antagonists. Biochem. Pharmacol..

[30] Hao S., Shi W., Liu W., Chen Q.Y., Zhuo M. (2023). Multiple modulatory roles of serotonin in chronic pain and injury-related anxiety. Front. Synaptic Neurosci..

[31] Satiamurthy R., Yaakob N.S., Shah N.M., Azmi N., Omar M.S. (2023). Potential Roles of 5-HT(3) Receptor Antagonists in Reducing Chemotherapy-induced Peripheral Neuropathy (CIPN). Curr. Mol. Med..

[32] Albert P.R., Vahid-Ansari F. (2019). The 5-HT1A receptor: Signaling to behavior. Biochimie.

[33] Haleem D.J. (2019). Targeting Serotonin1A Receptors for Treating Chronic Pain and Depression. Curr. Neuropharmacol..

[34] Vahid-Ansari F., Zhang M., Zahrai A., Albert P.R. (2019). Overcoming Resistance to Selective Serotonin Reuptake Inhibitors: Targeting Serotonin, Serotonin-1A Receptors and Adult Neuroplasticity. Front. Neurosci..

[35] Sarkar P., Mozumder S., Bej A., Mukherjee S., Sengupta J., Chattopadhyay A. (2020). Structure, dynamics and lipid interactions of serotonin receptors: Excitements and challenges. Biophys. Rev..

[36] Hayashida K.I., Obata H. (2019). Strategies to Treat Chronic Pain and Strengthen Impaired Descending Noradrenergic Inhibitory System. Int. J. Mol. Sci..

[37] Llorca-Torralba M., Borges G., Neto F., Mico J.A., Berrocoso E. (2016). Noradrenergic Locus Coeruleus pathways in pain modulation. Neuroscience.

[38] Caraci F., Merlo S., Drago F., Caruso G., Parenti C., Sortino M.A. (2019). Rescue of Noradrenergic System as a Novel Pharmacological Strategy in the Treatment of Chronic Pain: Focus on Microglia Activation. Front. Pharmacol..

[39] Obata H. (2017). Analgesic Mechanisms of Antidepressants for Neuropathic Pain. Int. J. Mol. Sci..

[40] Di Cesare Mannelli L., Micheli L., Crocetti L., Giovannoni M.P., Vergelli C., Ghelardini C. (2017). α2 Adrenoceptor: A Target for Neuropathic Pain Treatment. Mini Rev. Med. Chem..

[41] Inoue K. (2022). The Role of ATP Receptors in Pain Signaling. Neurochem. Res..

[42] Zou Y., Yang R., Li L., Xu X., Liang S. (2023). Purinergic signaling: A potential therapeutic target for depression and chronic pain. Purinergic Signal..

[43] Jung S.M., Peyton L., Essa H., Choi D.S. (2022). Adenosine receptors: Emerging non-opioids targets for pain medications. Neurobiol. Pain.

[44] Zhou M., Wu J., Chang H., Fang Y., Zhang D., Guo Y. (2023). Adenosine signaling mediate pain transmission in the central nervous system. Purinergic Signal..

[45] Sattler C., Benndorf K. (2022). Enlightening activation gating in P2X receptors. Purinergic Signal..

[46] Jacobson K.A., Pradhan B., Wen Z., Pramanik A. (2023). New paradigms in purinergic receptor ligand discovery. Neuropharmacology.

[47] Schrenk-Siemens K., Rösseler C., Lampert A. (2018). Translational Model Systems for Complex Sodium Channel Pathophysiology in Pain. Handb. Exp. Pharmacol..

[48] St John Smith E. (2018). Advances in understanding nociception and neuropathic pain. J. Neurol..

[49] Chen Y., Wu Q., Jin Z., Qin Y., Meng F., Zhao G. (2022). Review of Voltage-gated Calcium Channel α2δ Subunit Ligands for the Treatment of Chronic Neuropathic Pain and Insight into Structure-activity Relationship (SAR) by Pharmacophore Modeling. Curr. Med. Chem..

[50] Hoppanova L., Lacinova L. (2022). Voltage-dependent Ca(V)3.2 and Ca(V)2.2 channels in nociceptive pathways. Pflügers Arch..

[51] Abd-Elsayed A., Jackson M., Gu S.L., Fiala K., Gu J. (2019). Neuropathic pain and Kv7 voltage-gated potassium channels: The potential role of Kv7 activators in the treatment of neuropathic pain. Mol. Pain.

[52] Zemel B.M., Ritter D.M., Covarrubias M., Muqeem T. (2018). A-Type K(V) Channels in Dorsal Root Ganglion Neurons: Diversity, Function, and Dysfunction. Front. Mol. Neurosci..

[53] Li W.G., Xu T.L. (2015). Acid-sensing ion channels: A novel therapeutic target for pain and anxiety. Curr. Pharm. Des..

[54] Luo L., Song S., Ezenwukwa C.C., Jalali S., Sun B., Sun D. (2021). Ion channels and transporters in microglial function in physiology and brain diseases. Neurochem. Int..

[55] Valsecchi A.E., Franchi S., Panerai A.E., Rossi A., Sacerdote P., Colleoni M. (2011). The soy isoflavone genistein reverses oxidative and inflammatory state, neuropathic pain, neurotrophic and vasculature deficits in diabetes mouse model. Eur. J. Pharmacol..

[56] Hingtgen C.M., Waite K.J., Vasko M.R. (1995). Prostaglandins facilitate peptide release from rat sensory neurons by activating the adenosine 3′,5′-cyclic monophosphate transduction cascade. J. Neurosci..

[57] Page-McCaw A., Ewald A.J., Werb Z. (2007). Matrix metalloproteinases and the regulation of tissue remodelling. Nat. Rev. Mol. Cell Biol..

[58] Ji R.R., Xu Z.Z., Wang X., Lo E.H. (2009). Matrix metalloprotease regulation of neuropathic pain. Trends Pharmacol. Sci..

[59] Rahbardar M.G., Amin B., Mehri S., Mirnajafi-Zadeh S.J., Hosseinzadeh H. (2018). Rosmarinic acid attenuates development and existing pain in a rat model of neuropathic pain: An evidence of anti-oxidative and anti-inflammatory effects. Phytomedicine.

[60] Ghosh K., Pan H.L. (2022). Epigenetic Mechanisms of Neural Plasticity in Chronic Neuropathic Pain. ACS Chem. Neurosci..

[61] Luo D., Li X., Tang S., Song F., Li W., Xie G., Liang J., Zhou J. (2021). Epigenetic modifications in neuropathic pain. Mol. Pain.

[62] Zhang K., Li P., Jia Y., Liu M., Jiang J. (2022). Non-coding RNA and n6-methyladenosine modification play crucial roles in neuropathic pain. Front. Mol. Neurosci..

[63] Meaadi J., Obara I., Eldabe S., Nazar H. (2023). The safety and efficacy of gabapentinoids in the management of neuropathic pain: A systematic review with meta-analysis of randomised controlled trials. Int. J. Clin. Pharm..

[64] Cao X., Shen Z., Wang X., Zhao J., Liu W., Jiang G. (2023). A Meta-analysis of Randomized Controlled Trials Comparing the Efficacy and Safety of Pregabalin and Gabapentin in the Treatment of Postherpetic Neuralgia. Pain Ther..

[65] Giménez-Campos M.S., Pimenta-Fermisson-Ramos P., Díaz-Cambronero J.I., Carbonell-Sanchís R., López-Briz E., Ruíz-García V. (2022). A systematic review and meta-analysis of the effectiveness and adverse events of gabapentin and pregabalin for sciatica pain. Aten. Primaria.

[66] Shanthanna H., Gilron I., Rajarathinam M., AlAmri R., Kamath S., Thabane L., Devereaux P.J., Bhandari M. (2017). Benefits and safety of gabapentinoids in chronic low back pain: A systematic review and meta-analysis of randomized controlled trials. PLoS Med..

[67] Finnerup N.B., Attal N., Haroutounian S., McNicol E., Baron R., Dworkin R.H., Gilron I., Haanpää M., Hansson P., Jensen T.S. (2015). Pharmacotherapy for neuropathic pain in adults: A systematic review and meta-analysis. Lancet Neurol..

[68] Goins A., Patel K., Alles S.R.A. (2021). The gabapentinoid drugs and their abuse potential. Pharmacol. Ther..

[69] Bao H., Wu Z., Wang Q., Wang J., Zhang L., Meng L., Han F. (2021). The efficacy of gabapentin combined with opioids for neuropathic cancer pain: A meta-analysis. Transl. Cancer Res..

[70] Kremer M., Salvat E., Muller A., Yalcin I., Barrot M. (2016). Antidepressants and gabapentinoids in neuropathic pain: Mechanistic insights. Neuroscience.

[71] Sindrup S.H., Otto M., Finnerup N.B., Jensen T.S. (2005). Antidepressants in the treatment of neuropathic pain. Basic Clin. Pharmacol. Toxicol..

[72] Max M.B., Lynch S.A., Muir J., Shoaf S.E., Smoller B., Dubner R. (1992). Effects of desipramine, amitriptyline, and fluoxetine on pain in diabetic neuropathy. N. Engl. J. Med..

[73] Saarto T., Wiffen P.J. (2007). Antidepressants for neuropathic pain. Cochrane Database Syst. Rev..

[74] Attal N. (2019). Pharmacological treatments of neuropathic pain: The latest recommendations. Rev. Neurol..

[75] Lee Y.C., Chen P.P. (2010). A review of SSRIs and SNRIs in neuropathic pain. Expert. Opin. Pharmacother..

[76] Raskin J., Pritchett Y.L., Wang F., D’Souza D.N., Waninger A.L., Iyengar S., Wernicke J.F. (2005). A double-blind, randomized multicenter trial comparing duloxetine with placebo in the management of diabetic peripheral neuropathic pain. Pain Med..

[77] Goldstein D.J., Lu Y., Detke M.J., Lee T.C., Iyengar S. (2005). Duloxetine vs. placebo in patients with painful diabetic neuropathy. Pain.

[78] Wernicke J.F., Pritchett Y.L., D’Souza D.N., Waninger A., Tran P., Iyengar S., Raskin J. (2006). A randomized controlled trial of duloxetine in diabetic peripheral neuropathic pain. Neurology.

[79] Hossain S.M., Hussain S.M., Ekram A.R. (2016). Duloxetine in Painful Diabetic Neuropathy: A Systematic Review. Clin. J. Pain.

[80] Fornasari D., Magni A., Pais P., Palao T., Polati E., Sansone P. (2022). Changing the paradigm in postherpetic neuralgia treatment: Lidocaine 700 mg medicated plaster. Eur. Rev. Med. Pharmacol. Sci..

[81] Varshney V., Osborn J., Chaturvedi R., Shah V., Chakravarthy K. (2021). Advances in the interventional management of neuropathic pain. Ann. Transl. Med..

[82] Voute M., Morel V., Pickering G. (2021). Topical Lidocaine for Chronic Pain Treatment. Drug Des. Dev. Ther..

[83] Kocot-Kępska M., Zajączkowska R., Mika J., Kopsky D.J., Wordliczek J., Dobrogowski J., Przeklasa-Muszyńska A. (2021). Topical Treatments and Their Molecular/Cellular Mechanisms in Patients with Peripheral Neuropathic Pain-Narrative Review. Pharmaceutics.

[84] Gupta H., Patel A., Eswani Z., Moore P., Steib M., Lee C., Kaye A.D. (2021). Role of Intravenous Lidocaine Infusion in the Treatment of Peripheral Neuropathy. Orthop. Rev..

[85] Moulin D.E., Morley-Forster P.K., Pirani Z., Rohfritsch C., Stitt L. (2019). Intravenous lidocaine in the management of chronic peripheral neuropathic pain: A randomized-controlled trial. Can. J. Anaesth..

[86] Bnyan R., Khan I., Ehtezazi T., Saleem I., Gordon S., O’Neill F., Roberts M. (2019). Formulation and optimisation of novel transfersomes for sustained release of local anaesthetic. J. Pharm. Pharmacol..

[87] Attal N., Gaudé V., Brasseur L., Dupuy M., Guirimand F., Parker F., Bouhassira D. (2000). Intravenous lidocaine in central pain: A double-blind, placebo-controlled, psychophysical study. Neurology.

[88] Fan Z., Zheng X., Li D., Chen H., Li L. (2022). Comparison of lidocaine and ropivacaine stellate ganglion blockade in treating upper limb postherpetic neuralgia. Medicine.

[89] Jang Y.J., Lee J.H., Seo T.B., Oh S.H. (2017). Lidocaine/multivalent ion complex as a potential strategy for prolonged local anesthesia. Eur. J. Pharm. Biopharm..

[90] Jiang J., Wu H., Zou Z. (2022). In vitro and in vivo evaluation of a novel lidocaine-loaded cubosomal gel for prolonged local anesthesia. J. Biomater. Appl..

[91] Maulvi F.A., Pillai L.V., Patel K.P., Desai A.R., Shukla M.R., Desai D.T., Patel H.P., Ranch K.M., Shah S.A., Shah D.O. (2020). Lidocaine tripotassium phosphate complex laden microemulsion for prolonged local anaesthesia: In vitro and in vivo studies. Colloids Surf. B Biointerfaces.

[92] Xu X., Chang S., Zhang X., Hou T., Yao H., Zhang S., Zhu Y., Cui X., Wang X. (2022). Fabrication of a controlled-release delivery system for relieving sciatica nerve pain using an ultrasound-responsive microcapsule. Front. Bioeng. Biotechnol..

[93] Baron R., Mayoral V., Leijon G., Binder A., Steigerwald I., Serpell M. (2009). Efficacy and safety of 5% lidocaine (lignocaine) medicated plaster in comparison with pregabalin in patients with postherpetic neuralgia and diabetic polyneuropathy: Interim analysis from an open-label, two-stage adaptive, randomized, controlled trial. Clin. Drug Investig..

[94] Maloney J., Pew S., Wie C., Gupta R., Freeman J., Strand N. (2021). Comprehensive Review of Topical Analgesics for Chronic Pain. Curr. Pain Headache Rep..

[95] Srinivasan K. (2016). Biological Activities of Red Pepper (*Capsicum annuum*) and Its Pungent Principle Capsaicin: A Review. Crit. Rev. Food Sci. Nutr..

[96] Fernandes E.S., Cerqueira A.R., Soares A.G., Costa S.K. (2016). Capsaicin and Its Role in Chronic Diseases. Adv. Exp. Med. Biol..

[97] Bonezzi C., Costantini A., Cruccu G., Fornasari D.M.M., Guardamagna V., Palmieri V., Polati E., Zini P., Dickenson A.H. (2020). Capsaicin 8% dermal patch in clinical practice: An expert opinion. Expert. Opin. Pharmacother..

[98] Giaccari L.G., Aurilio C., Coppolino F., Pace M.C., Passsavanti M.B., Pota V., Sansone P. (2021). Capsaicin 8% Patch and Chronic Postsurgical Neuropathic Pain. J. Pers. Med..

[99] Leavell Y., Simpson D.M. (2022). The role of the capsaicin 8% patch in the treatment of painful diabetic peripheral neuropathy. Pain Manag..

[100] Olusanya A., Yearsley A., Brown N., Braun S., Hayes C., Rose E., Connolly B., Dicks M., Beal C., Helmonds B. (2023). Capsaicin 8% Patch for Spinal Cord Injury Focal Neuropathic Pain, a Randomized Controlled Trial. Pain Med..

[101] Cabezón-Gutiérrez L., Custodio-Cabello S., Palka-Kotlowska M., Khosravi-Shahi P. (2020). High-Dose 8% Capsaicin Patch in Treatment of Chemotherapy-Induced Peripheral Neuropathy. A Systematic Review. J. Pain Symptom Manag..

[102] Dosenovic S., Jelicic Kadic A., Miljanovic M., Biocic M., Boric K., Cavar M., Markovina N., Vucic K., Puljak L. (2017). Interventions for Neuropathic Pain: An Overview of Systematic Reviews. Anesth. Analg..

[103] Bates D., Schultheis B.C., Hanes M.C., Jolly S.M., Chakravarthy K.V., Deer T.R., Levy R.M., Hunter C.W. (2019). A Comprehensive Algorithm for Management of Neuropathic Pain. Pain Med..

[104] Muthuraman A., Singh N., Jaggi A.S., Ramesh M. (2014). Drug therapy of neuropathic pain: Current developments and future perspectives. Curr. Drug Targets.

[105] Yang Y., Maher D.P., Cohen S.P. (2020). Emerging concepts on the use of ketamine for chronic pain. Expert. Rev. Clin. Pharmacol..

[106] Israel J.E., St Pierre S., Ellis E., Hanukaai J.S., Noor N., Varrassi G., Wells M., Kaye A.D. (2021). Ketamine for the Treatment of Chronic Pain: A Comprehensive Review. Health Psychol. Res..

[107] Obeng S., Hiranita T., León F., McMahon L.R., McCurdy C.R. (2021). Novel Approaches, Drug Candidates, and Targets in Pain Drug Discovery. J. Med. Chem..

[108] Taneja A., Della Pasqua O., Danhof M. (2017). Challenges in translational drug research in neuropathic and inflammatory pain: The prerequisites for a new paradigm. Eur. J. Clin. Pharmacol..

[109] Vo T., Rice A.S.C., Dworkin R.H. (2009). Non-steroidal anti-inflammatory drugs for neuropathic pain: How do we explain continued widespread use?. Pain.

[110] Boyd A., Bleakley C., Hurley D.A., Gill C., Hannon-Fletcher M., Bell P., McDonough S. (2019). Herbal medicinal products or preparations for neuropathic pain. Cochrane Database Syst. Rev..

[111] Jahromi B., Pirvulescu I., Candido K.D., Knezevic N.N. (2021). Herbal Medicine for Pain Management: Efficacy and Drug Interactions. Pharmaceutics.

[112] Gaskell H., Derry S., Stannard C., Moore R.A. (2016). Oxycodone for neuropathic pain in adults. Cochrane Database Syst. Rev..

[113] Alshehri F.S. (2023). Tapentadol: A Review of Experimental Pharmacology Studies, Clinical Trials, and Recent Findings. Drug Des. Dev. Ther..

[114] Barakat A. (2019). Revisiting Tramadol: A Multi-Modal Agent for Pain Management. CNS Drugs.

[115] Faria J., Barbosa J., Moreira R., Queirós O., Carvalho F., Dinis-Oliveira R.J. (2018). Comparative pharmacology and toxicology of tramadol and tapentadol. Eur. J. Pain.

[116] Sisignano M., Gribbon P., Geisslinger G. (2022). Drug Repurposing to Target Neuroinflammation and Sensory Neuron-Dependent Pain. Drugs.

[117] Cazan D., Klimek L., Sperl A., Plomer M., Kölsch S. (2018). Safety of ambroxol in the treatment of airway diseases in adult patients. Expert. Opin. Drug Saf..

[118] Malerba M., Ragnoli B. (2008). Ambroxol in the 21st century: Pharmacological and clinical update. Expert. Opin. Drug Metab. Toxicol..

[119] Russo M.A., Baron R., Dickenson A.H., Kern K.U., Santarelli D.M. (2023). Ambroxol for neuropathic pain: Hiding in plain sight?. Pain.

[120] Salat K., Gryzlo B., Kulig K. (2018). Experimental Drugs for Neuropathic Pain. Curr. Neuropharmacol..

[121] Gaida W., Klinder K., Arndt K., Weiser T. (2005). Ambroxol, a Nav1.8-preferring Na^+^ channel blocker, effectively suppresses pain symptoms in animal models of chronic, neuropathic and inflammatory pain. Neuropharmacology.

[122] Furgała A., Fijałkowski Ł., Nowaczyk A., Sałat R., Sałat K. (2018). Time-shifted co-administration of sub-analgesic doses of ambroxol and pregabalin attenuates oxaliplatin-induced cold allodynia in mice. Biomed. Pharmacother..

[123] Hama A.T., Plum A.W., Sagen J. (2010). Antinociceptive effect of ambroxol in rats with neuropathic spinal cord injury pain. Pharmacol. Biochem. Behav..

[124] Kern K.U., Weiser T. (2015). Topical ambroxol for the treatment of neuropathic pain. An initial clinical observation. Schmerz.

[125] Maihöfner C., Schneider S., Bialas P., Gockel H., Beer K.G., Bartels M., Kern K.U. (2018). Successful treatment of complex regional pain syndrome with topical ambroxol: A case series. Pain Manag..

[126] Kern K.U., Schwickert-Nieswandt M., Maihöfner C., Gaul C. (2019). Topical Ambroxol 20% for the Treatment of Classical Trigeminal Neuralgia—A New Option? Initial Clinical Case Observations. Headache.

[127] McCarberg B.H., Barkin R.L. (2007). The future of cannabinoids as analgesic agents: A pharmacologic, pharmacokinetic, and pharmacodynamic overview. Am. J. Ther..

[128] Karst M., Salim K., Burstein S., Conrad I., Hoy L., Schneider U. (2003). Analgesic effect of the synthetic cannabinoid CT-3 on chronic neuropathic pain: A randomized controlled trial. JAMA.

[129] Atakan Z. (2012). Cannabis, a complex plant: Different compounds and different effects on individuals. Ther. Adv. Psychopharmacol..

[130] Kogan N.M., Mechoulam R. (2007). Cannabinoids in health and disease. Dialogues Clin. Neurosci..

[131] Malfait A.M., Gallily R., Sumariwalla P.F., Malik A.S., Andreakos E., Mechoulam R., Feldmann M. (2000). The nonpsychoactive cannabis constituent cannabidiol is an oral anti-arthritic therapeutic in murine collagen-induced arthritis. Proc. Natl. Acad. Sci. USA.

[132] Hampson A.J., Grimaldi M., Axelrod J., Wink D. (1998). Cannabidiol and (-)Delta9-tetrahydrocannabinol are neuroprotective antioxidants. Proc. Natl. Acad. Sci. USA.

[133] Rock E.M., Bolognini D., Limebeer C.L., Cascio M.G., Anavi-Goffer S., Fletcher P.J., Mechoulam R., Pertwee R.G., Parker L.A. (2012). Cannabidiol, a non-psychotropic component of cannabis, attenuates vomiting and nausea-like behaviour via indirect agonism of 5-HT(1A) somatodendritic autoreceptors in the dorsal raphe nucleus. Br. J. Pharmacol..

[134] Baker D., Pryce G., Croxford J.L., Brown P., Pertwee R.G., Huffman J.W., Layward L. (2000). Cannabinoids control spasticity and tremor in a multiple sclerosis model. Nature.

[135] Shahbazi F., Grandi V., Banerjee A., Trant J.F. (2020). Cannabinoids and Cannabinoid Receptors: The Story so Far. iScience.

[136] Pertwee R.G. (2008). The diverse CB1 and CB2 receptor pharmacology of three plant cannabinoids: Delta9-tetrahydrocannabinol, cannabidiol and delta9-tetrahydrocannabivarin. Br. J. Pharmacol..

[137] Oláh A., Szekanecz Z., Bíró T. (2017). Targeting Cannabinoid Signaling in the Immune System: “High”-ly Exciting Questions, Possibilities, and Challenges. Front. Immunol..

[138] Burstein S. (2015). Cannabidiol (CBD) and its analogs: A review of their effects on inflammation. Bioorganic Med. Chem..

[139] Elmes M.W., Kaczocha M., Berger W.T., Leung K., Ralph B.P., Wang L., Sweeney J.M., Miyauchi J.T., Tsirka S.E., Ojima I. (2015). Fatty acid-binding proteins (FABPs) are intracellular carriers for Δ9-tetrahydrocannabinol (THC) and cannabidiol (CBD). J. Biol. Chem..

[140] Petzke F., Tölle T., Fitzcharles M.A., Häuser W. (2022). Cannabis-Based Medicines and Medical Cannabis for Chronic Neuropathic Pain. CNS Drugs.

[141] Campos R.M.P., Aguiar A.F.L., Paes-Colli Y., Trindade P.M.P., Ferreira B.K., de Melo Reis R.A., Sampaio L.S. (2021). Cannabinoid Therapeutics in Chronic Neuropathic Pain: From Animal Research to Human Treatment. Front. Physiol..

[142] Baron-Flores V., Diaz-Ruiz A., Manzanares J., Rios C., Burelo M., Jardon-Guadarrama G., Martínez-Cárdenas M., Mata-Bermudez A. (2022). Cannabidiol attenuates hypersensitivity and oxidative stress after traumatic spinal cord injury in rats. Neurosci. Lett..

[143] Dos Santos R., Veras F., Netto G., Elisei L., Sorgi C., Faccioli L., Galdino G. (2023). Cannabidiol prevents chemotherapy-induced neuropathic pain by modulating spinal TLR4 via endocannabinoid system activation. J. Pharm. Pharmacol..

[144] Eeswara A., Pacheco-Spiewak A., Jergova S., Sagen J. (2023). Combined non-psychoactive Cannabis components cannabidiol and β-caryophyllene reduce chronic pain via CB1 interaction in a rat spinal cord injury model. PLoS ONE.

[145] Haleem R., Wright R. (2020). A Scoping Review on Clinical Trials of Pain Reduction With Cannabis Administration in Adults. J. Clin. Med. Res..

[146] Sainsbury B., Bloxham J., Pour M.H., Padilla M., Enciso R. (2021). Efficacy of cannabis-based medications compared to placebo for the treatment of chronic neuropathic pain: A systematic review with meta-analysis. J. Dent. Anesth. Pain Med..

[147] Filippini G., Minozzi S., Borrelli F., Cinquini M., Dwan K. (2022). Cannabis and cannabinoids for symptomatic treatment for people with multiple sclerosis. Cochrane Database Syst. Rev..

[148] Arout C.A., Haney M., Herrmann E.S., Bedi G., Cooper Z.D. (2022). A placebo-controlled investigation of the analgesic effects, abuse liability, safety and tolerability of a range of oral cannabidiol doses in healthy humans. Br. J. Clin. Pharmacol..

[149] Chesney E., Oliver D., Green A., Sovi S., Wilson J., Englund A., Freeman T.P., McGuire P. (2020). Adverse effects of cannabidiol: A systematic review and meta-analysis of randomized clinical trials. Neuropsychopharmacology.

[150] Ford T.C., Hayley A.C., Downey L.A., Parrott A.C. (2017). Cannabis: An Overview of its Adverse Acute and Chronic Effects and its Implications. Curr. Drug Abus. Rev..

[151] Huestis M.A., Solimini R., Pichini S., Pacifici R., Carlier J., Busardò F.P. (2019). Cannabidiol Adverse Effects and Toxicity. Curr. Neuropharmacol..

[152] Lucas C.J., Galettis P., Schneider J. (2018). The pharmacokinetics and the pharmacodynamics of cannabinoids. Br. J. Clin. Pharmacol..

[153] Huestis M.A. (2007). Human cannabinoid pharmacokinetics. Chem. Biodivers..

[154] Bakare A.O., Owoyele B.V. (2020). Antinociceptive and neuroprotective effects of bromelain in chronic constriction injury-induced neuropathic pain in Wistar rats. Korean J. Pain.

[155] Bakare A.O., Owoyele B.V. (2020). Bromelain reversed electrolyte imbalance in the chronically constricted sciatic nerve of Wistar rats. Naunyn Schmiedebergs Arch. Pharmacol..

[156] Bakare A.O., Owoyele B.V. (2021). Bromelain reduced pro-inflammatory mediators as a common pathway that mediate antinociceptive and anti-anxiety effects in sciatic nerve ligated Wistar rats. Sci. Rep..

[157] Desideri I., Francolini G., Becherini C., Terziani F., Delli Paoli C., Olmetto E., Loi M., Perna M., Meattini I., Scotti V. (2017). Use of an alpha lipoic, methylsulfonylmethane and bromelain dietary supplement (Opera(^®^)) for chemotherapy-induced peripheral neuropathy management, a prospective study. Med. Oncol..

[158] Carpentieri A., Díaz de Barboza G., Areco V., Peralta López M., Tolosa de Talamoni N. (2012). New perspectives in melatonin uses. Pharmacol. Res..

[159] Hardeland R., Cardinali D.P., Srinivasan V., Spence D.W., Brown G.M., Pandi-Perumal S.R. (2011). Melatonin—A pleiotropic, orchestrating regulator molecule. Prog. Neurobiol..

[160] Acuña Castroviejo D., López L.C., Escames G., López A., García J.A., Reiter R.J. (2011). Melatonin-mitochondria interplay in health and disease. Curr. Top. Med. Chem..

[161] Reiter R.J., Mayo J.C., Tan D.X., Sainz R.M., Alatorre-Jimenez M., Qin L. (2016). Melatonin as an antioxidant: Under promises but over delivers. J. Pineal Res..

[162] López A., García J.A., Escames G., Venegas C., Ortiz F., López L.C., Acuña-Castroviejo D. (2009). Melatonin protects the mitochondria from oxidative damage reducing oxygen consumption, membrane potential, and superoxide anion production. J. Pineal Res..

[163] Leon J., Acuña-Castroviejo D., Sainz R.M., Mayo J.C., Tan D.X., Reiter R.J. (2004). Melatonin and mitochondrial function. Life Sci..

[164] Acuña-Castroviejo D., Martín M., Macías M., Escames G., León J., Khaldy H., Reiter R.J. (2001). Melatonin, mitochondria, and cellular bioenergetics. J. Pineal Res..

[165] Liu L., Labani N., Cecon E., Jockers R. (2019). Melatonin Target Proteins: Too Many or Not Enough?. Front. Endocrinol..

[166] Legros C., Chesneau D., Boutin J.A., Barc C., Malpaux B. (2014). Melatonin from cerebrospinal fluid but not from blood reaches sheep cerebral tissues under physiological conditions. J. Neuroendocrinol..

[167] Kuthati Y., Lin S.H., Chen I.J., Wong C.S. (2019). Melatonin and their analogs as a potential use in the management of Neuropathic pain. J. Formos. Med. Assoc..

[168] Srinivasan V., Lauterbach E.C., Ho K.Y., Acuña-Castroviejo D., Zakaria R., Brzezinski A. (2012). Melatonin in antinociception: Its therapeutic applications. Curr. Neuropharmacol..

[169] Ambriz-Tututi M., Rocha-González H.I., Cruz S.L., Granados-Soto V. (2009). Melatonin: A hormone that modulates pain. Life Sci..

[170] Srinivasan V., Zakaria R., Jeet Singh H., Acuna-Castroviejo D. (2012). Melatonin and its agonists in pain modulation and its clinical application. Arch. Ital. Biol..

[171] Posa L., De Gregorio D., Gobbi G., Comai S. (2018). Targeting Melatonin MT2 Receptors: A Novel Pharmacological Avenue for Inflammatory and Neuropathic Pain. Curr. Med. Chem..

[172] Al-Omary F.A. (2013). Melatonin: Comprehensive profile. Profiles Drug Subst. Excip. Relat. Methodol..

[173] Dai C.Q., Guo Y., Chu X.Y. (2020). Neuropathic Pain: The Dysfunction of Drp1, Mitochondria, and ROS Homeostasis. Neurotox. Res..

[174] Landis C.A. (2014). Is melatonin the next “new” therapy to improve sleep and reduce pain?. Sleep.

[175] Borsani E., Buffoli B., Bonazza V., Reiter R.J., Rezzani R., Rodella L.F. (2017). Single Administration of Melatonin Modulates the Nitroxidergic System at the Peripheral Level and Reduces Thermal Nociceptive Hypersensitivity in Neuropathic Rats. Int. J. Mol. Sci..

[176] Huang C.T., Chen S.H., Chang C.F., Lin S.C., Lue J.H., Tsai Y.J. (2020). Melatonin reduces neuropathic pain behavior and glial activation through MT(2) melatonin receptor modulation in a rat model of lysophosphatidylcholine-induced demyelination neuropathy. Neurochem. Int..

[177] Fakhri S., Kiani A., Jalili C., Abbaszadeh F., Piri S., Farzaei M.H., Rastegari-Pouyani M., Mohammadi-Noori E., Khan H. (2021). Intrathecal Administration of Melatonin Ameliorates the Neuroinflammation- Mediated Sensory and Motor Dysfunction in A Rat Model of Compression Spinal Cord Injury. Curr. Mol. Pharmacol..

[178] Mokhtari T., Yue L.P., Hu L. (2023). Exogenous melatonin alleviates neuropathic pain-induced affective disorders by suppressing NF-κB/ NLRP3 pathway and apoptosis. Sci. Rep..

[179] Marchesi N., Govoni S., Allegri M. (2022). Non-drug pain relievers active on non-opioid pain mechanisms. Pain Pract..

[180] Raghu G., Berk M., Campochiaro P.A., Jaeschke H., Marenzi G., Richeldi L., Wen F.Q., Nicoletti F., Calverley P.M.A. (2021). The Multifaceted Therapeutic Role of N-Acetylcysteine (NAC) in Disorders Characterized by Oxidative Stress. Curr. Neuropharmacol..

[181] Horst A., de Souza J.A., Santos M.C.Q., Riffel A.P.K., Kolberg C., Partata W.A. (2017). Effects of N-acetylcysteine on spinal cord oxidative stress biomarkers in rats with neuropathic pain. Braz. J. Med. Biol. Res..

[182] Horst A., Kolberg C., Moraes M.S., Riffel A.P., Finamor I.A., Belló-Klein A., Pavanato M.A., Partata W.A. (2014). Effect of N-acetylcysteine on the spinal-cord glutathione system and nitric-oxide metabolites in rats with neuropathic pain. Neurosci. Lett..

[183] Bernabucci M., Notartomaso S., Zappulla C., Fazio F., Cannella M., Motolese M., Battaglia G., Bruno V., Gradini R., Nicoletti F. (2012). N-Acetyl-cysteine causes analgesia by reinforcing the endogenous activation of type-2 metabotropic glutamate receptors. Mol. Pain.

[184] Özgül C., Nazıroğlu M. (2012). TRPM2 channel protective properties of N-acetylcysteine on cytosolic glutathione depletion dependent oxidative stress and Ca^2+^ influx in rat dorsal root ganglion. Physiol. Behav..

[185] Sözbir E., Nazıroğlu M. (2016). Diabetes enhances oxidative stress-induced TRPM2 channel activity and its control by N-acetylcysteine in rat dorsal root ganglion and brain. Metab. Brain Dis..

[186] Notartomaso S., Scarselli P., Mascio G., Liberatore F., Mazzon E., Mammana S., Gugliandolo A., Cruccu G., Bruno V., Nicoletti F. (2020). N-Acetylcysteine causes analgesia in a mouse model of painful diabetic neuropathy. Mol. Pain.

[187] Li J., Xu L., Deng X., Jiang C., Pan C., Chen L., Han Y., Dai W., Hu L., Zhang G. (2016). N-acetyl-cysteine attenuates neuropathic pain by suppressing matrix metalloproteinases. Pain.

[188] Zhu D., Fan T., Chen Y., Huo X., Li Y., Liu D., Cai Y., Cheung C.W., Tang J., Cui J. (2022). CXCR4/CX43 Regulate Diabetic Neuropathic Pain via Intercellular Interactions between Activated Neurons and Dysfunctional Astrocytes during Late Phase of Diabetes in Rats and the Effects of Antioxidant N-Acetyl-L-Cysteine. Oxid. Med. Cell Longev..

[189] Heidari N., Sajedi F., Mohammadi Y., Mirjalili M., Mehrpooya M. (2019). Ameliorative Effects Of N-Acetylcysteine As Adjunct Therapy On Symptoms Of Painful Diabetic Neuropathy. J. Pain Res..

[190] Mohiuddin M., Pivetta B., Gilron I., Khan J.S. (2021). Efficacy and Safety of N-Acetylcysteine for the Management of Chronic Pain in Adults: A Systematic Review and Meta-Analysis. Pain Med..

[191] Santos W., Guimarães J.O., Pina L.T.S., Serafini M.R., Guimarães A.G. (2022). Antinociceptive effect of plant-based natural products in chemotherapy-induced peripheral neuropathies: A systematic review. Front. Pharmacol..

[192] Freo U., Brugnatelli V., Turco F., Zanette G. (2021). Analgesic and Antidepressant Effects of the Clinical Glutamate Modulators Acetyl-L-Carnitine and Ketamine. Front. Neurosci..

[193] Sarzi-Puttini P., Giorgi V., Di Lascio S., Fornasari D. (2021). Acetyl-L-carnitine in chronic pain: A narrative review. Pharmacol. Res..

[194] Rolim L.C., da Silva E.M., Flumignan R.L., Abreu M.M., Dib S.A. (2019). Acetyl-L-carnitine for the treatment of diabetic peripheral neuropathy. Cochrane Database Syst. Rev..

[195] Rowin J. (2019). Integrative neuromuscular medicine: Neuropathy and neuropathic pain: Consider the alternatives. Muscle Nerve.

[196] Viana M.D.M., Lauria P.S.S., Lima A.A., Opretzka L.C.F., Marcelino H.R., Villarreal C.F. (2022). Alpha-Lipoic Acid as an Antioxidant Strategy for Managing Neuropathic Pain. Antioxidants.

[197] Lang-Illievich K., Klivinyi C., Lasser C., Brenna C.T.A., Szilagyi I.S., Bornemann-Cimenti H. (2023). Palmitoylethanolamide in the Treatment of Chronic Pain: A Systematic Review and Meta-Analysis of Double-Blind Randomized Controlled Trials. Nutrients.

[198] Yousefi-Manesh H., Shirooie S., Noori T., Sheibani M., Tavangar S.M., Hemmati S., Sadeghi M.A., Akbarniakhaky H., Mohammadi Z., Foroutani L. (2023). Spermidine reduced neuropathic pain in chronic constriction injury-induced peripheral neuropathy in rats. Fundam. Clin. Pharmacol..

[199] Miguel C.A., Noya-Riobó M.V., Mazzone G.L., Villar M.J., Coronel M.F. (2021). Antioxidant, anti-inflammatory and neuroprotective actions of resveratrol after experimental nervous system insults. Special focus on the molecular mechanisms involved. Neurochem. Int..

[200] Shen C.L., Castro L., Fang C.Y., Castro M., Sherali S., White S., Wang R., Neugebauer V. (2022). Bioactive compounds for neuropathic pain: An update on preclinical studies and future perspectives. J. Nutr. Biochem..

[201] Sun J., Chen F., Braun C., Zhou Y.Q., Rittner H., Tian Y.K., Cai X.Y., Ye D.W. (2018). Role of curcumin in the management of pathological pain. Phytomedicine.

[202] Urošević M., Nikolić L., Gajić I., Nikolić V., Dinić A., Miljković V. (2022). Curcumin: Biological Activities and Modern Pharmaceutical Forms. Antibiotics.

[203] Roganović J., Petrović N. (2022). Clinical Perspectives of Non-Coding RNA in Oral Inflammatory Diseases and Neuropathic Pain: A Narrative Review. Int. J. Mol. Sci..

[204] Reis C., Chambel S., Ferreira A., Cruz C.D. (2023). Involvement of nerve growth factor (NGF) in chronic neuropathic pain—A systematic review. Rev. Neurosci..

